# Mechanical properties of animal ligaments: a review and comparative study for the identification of the most suitable human ligament surrogates

**DOI:** 10.1007/s10237-023-01718-1

**Published:** 2023-05-11

**Authors:** V. Burgio, S. Casari, M. Milizia, F. Sanna, G. Spezia, M. Civera, M. Rodriguez Reinoso, A. Bertuglia, C. Surace

**Affiliations:** 1 Department of Structural, Building and Geotechnical Engineering, Politecnico di Torino, 10129 Turin, Italy; 2 Department of Structural, Geotechnical and Building Engineering, Laboratory of Bio-Inspired Nanomechanics, Politecnico di Torino, Corso Duca Degli Abruzzi 24, 10129 Turin, Italy; 3 Department of Veterinary Science, University of Turin, Largo Paolo Braccini 2-5, 10095 Grugliasco, Italy

**Keywords:** Animal ligaments, Human ligaments, Mechanical properties, Tensile properties, Mechanical characterisation, Biomechanics

## Abstract

The interest in the properties of animal soft tissues is often related to the desire to find an animal model to replace human counterparts due to the unsteady availability of human tissues for experimental purposes. Once the most appropriate animal model is identified, it is possible to carry out ex-vivo and in-vivo studies for the repair of ligamentous tissues and performance testing of replacement and support healing devices. This work aims to present a systematic review of the mechanical properties of ligaments reported in the scientific literature by considering different anatomical regions in humans and several animal species. This study was conducted according to the Preferred Reporting Items for Systematic Reviews and Meta-Analyses (PRISMA) method. Moreover, considering the lack of a standard protocol for preconditioning of tissues, this aspect is also addressed. Ninety-six studies were selected for the systematic review and analysed. The mechanical properties of different animal species are reported and summarised in tables. Only results from studies reporting the strain rate parameter were considered for comparison with human ligaments, as they were deemed more reliable. Elastic modulus, ultimate tensile stress, and ultimate strain properties are graphically reported identifying the range of values for each animal species and to facilitate comparison between values reported in the scientific literature in animal and human ligaments. Useful similarities between the mechanical properties of swine, cow, and rat and human ligaments have been found.

**Supplementary Information:**

The online version contains supplementary material available at 10.1007/s10237-023-01718-1.

## Introduction

The interest in the mechanical properties of animal ligaments is often correlated with finding a useful model for human ones. Since ethical reasons make difficult to find human ligaments to run in vitro and in vivo tests, animal specimens are commonly employed. In fact, animal models are preferred in preclinical studies for two main types of research purposes: (i) evaluation of tissue healing through different strategies (for example, after growth factors and stem cell injection) and (ii) the evaluation of mechanical properties of suture pattern under validation and testing of innovative repair technologies. Surgical repair techniques commonly employed in human’s and animal’s traumatology (DeLong and Waterman 2015; Dabbene et al. 2018) rely on the results of mechanical studies, based on reported properties of the original and intact anatomical structures.

Nevertheless, not all animal ligaments are biomechanically comparable to their humans’ anatomical counterparts. Therefore, it is needed to discuss the differences between these latter and animal ligaments, even if few studies in the literature made a direct comparison between human and animal ligaments (Baah-Dwomoh et al. 2018; Noyes and Grood 1976).

This review aims to provide a more detailed analysis of similarities and differences between human and various animal species to find the most suitable human ligament surrogate. Uniaxial tensile tests performed on equine, bovine, ovine, caprine, swine, canine, rodents, leporidae, and human ligaments were considered. This work is closely related to a similar comparison between animal and human tendons previously conducted by this research group (Burgio et al. 2022).

### Human and animal ligaments

Like tendons, ligaments are characterised by a hierarchical structure and are made of mesenchymal cells inside a supporting matrix and an extracellular matrix containing a high amount of collagen fibres (type I and type III collagen are the most abundant), water and to a lesser extent of elastin, glycoproteins, and proteoglycans (Rumian et al. 2007).

Despite the similar composition, in tendons collagen fibrils are placed in parallel to each other and along the whole length of the tendon. On the contrary, the collagen fibrils of the ligaments are not uniformly orientated, and this organisation is fundamental to withstand multidirectional loads (Rumian et al. 2007).

Even localisation and function, as well as the different arrangement of the components, contribute to defining differences in the biomechanical characteristics of tendons and ligaments: both of these structures must be able to withstand tensile loads, but while the tendons are subjected mostly to uniaxial forces, the ligaments are subjected to multiaxial loads (the force components directions depend on the directions of movement allowed to the joint) (Rumian et al. 2007).

### Common applications of animal surrogates

Concerning biomechanics, it is important to consider that, unlike humans, almost all animals are quadrupeds and often have different and more limited ranges of motion in the corresponding joints (Bascuñán et al. 2019). However, there are many instances where they are extensively used. In this section, will be discussed different animal models encountered in the research. Considering human biomechanics, the main subject of investigation is the knee joint; therefore, over the years several studies with different animal models have been done to better understand its anatomy and biomechanics. On the other hand, only a few articles dealing with other anatomical sites were found, and these will be discussed in a specific subsection.

#### Animal models for knee joint

Knee joint ligaments injuries are one of the most widespread lesions; for this reason, several animal models have been widely employed to better understand the anatomy and biomechanics. Numerous studies dealing with knee ligament reconstruction via suture patterns, graft, or Ligament Advanced Reinforcement System (LARS) used animal specimens to perform tests, especially bovine (Eleswarapu et al. 2011), rabbit (Woo et al. 1992), rat (Yiannakopoulos et al. 2005), sheep (Weiler et al. 2001; Viateau et al. 2013), swine (Kim et al. 2014), and monkey (Noyes and Grood 1976). To the best of our knowledge, the study carried out by Noyes and Grood (Noyes and Grood 1976) is the only one in the literature that deals with a nonquadruped animal model, and the authors reported similar results with respect to the canine model.

The anterior cruciate ligament (ACL) is critical for knee joint stability in humans and animals, and its injury results in joint instability rapidly causing osteoarthritis (Comerford et al. 2005). The canine knee model is largely used to make studies on knee ligaments and tendons due to its similarity with its human counterpart (Beynnon et al. 1994).

The sheep stifle joint has often been used as an animal model for human ACL reconstruction. However, Radford et al. (1996), showed that the ovine stifle is not suitable for testing full-size human clinical ACL implants. The reason for this statement is that when compared to human joints the overall shape of the distal femur is narrower, and the femoral condyles do not have extensive articular surfaces distally. Thus, the range of motion of the stifle is not adapted for taking loads in full extension and cannot attain a straight-leg posture (Radford et al. 1996).

Moreover, it was concluded that the stifle joint of the sheep is both morphologically and biomechanically similar to the human knee, but there are detailed differences relating to ligament’s fibres geometry. In conclusion, the authors reported that the ovine stifle is a valid animal model for experimental work on menisci and cruciate ligaments (Radford et al. 1996).

The rabbit knee has often been used as an animal model for the study of cruciate ligaments (posterior cruciate ligament (PCL) and ACL) and collateral ligaments (medial collateral ligament (MCL) and the lateral collateral ligament (LCL)). It is well accepted in the orthopaedic community that unrepaired injuries to either cruciate ligament will eventually result in chronic secondary degenerative joint changes, most notably in the menisci and in the articular cartilage. Few studies have been proposed to analyse the pathological consequences of cruciate ligament ruptures in the medial and collateral ligaments. Among them, Tozilli and Arnoczky (Tozilli and Arnoczky 1988) have not found significant changes in the biomechanical properties of rabbit LCL after a complete section of the anterior and posterior cruciate ligaments.

Another knee ligament involved in common trauma is the MCL; therefore, it is of great importance to find suitable animal surrogates. A relevant case study was conducted by Germscheid et al. (2011), in which was reported that porcine MCL is comparable in shape and size and in its failure mechanism to the adult human MCL.

#### Other animal models

Animal models are often used also to investigate causes and consequences of human diseases on the related ligaments. For example, a frequent trauma highly explored is the chronic neck pain caused by whiplash; in this context, several tensile failure studies (Lee et al. 2006; Quinn and Winkelstein 2007) of the C6/C7 rat cervical facet capsular ligament have been conducted to better understand the whiplash-related pain. Other studies were also conducted to better understand pelvic floor disorders that often result on permanent compromission of pelvic ligaments, affecting millions of women every year. The pelvic anatomy of the Macaca species is approximately identical to that of the human, providing a unique opportunity to study pelvic supportive ligaments (Vardy et al. 2005) and related mechanical and structural changes after injuries. Studied on non-quadruped animals which have a certain relevance, since they have a posture and joint range of motion more similar to that of humans. Unfortunately, in our research work, only one study on non-quadrupeds animals met the eligibility criteria and therefore was considered worthy of being reviewed. The results obtained are interesting, and comparisons with human ligaments have been performed in paragraph 4.1.1.

### Effects of experimental setup parameters

First of all, it is necessary to specify that to characterise the ligaments and evaluate the integrity of the tissues after surgical repair, uniaxial tensile tests are generally carried out on the bone–ligament–bone (blb) complexes rather than on the single, isolated ligament. This procedure is preferred due to the limited sizes of the single ligament and its slipperiness at the anchor points with the clamps. The bone provides a secure hold on clamps during in-vitro testing. In contrast, the blb complex has one drawback: often the break occurs near the insertions (avulsion) instead of the expected “mid-substance failure” (Sample 2017; Martin et al. 2015).

Due to the variability in the ligament’s mechanical properties introduced by the animal species, age, sex, testing conditions, tensile testing device and orientation of the ligaments or blb complexes in relation to the imposed stress, it is crucial to standardise a protocol to obtain data easily comparable with each other (Beynnon and Amis 1998). In this systematic review, wherever available, these parameters are always reported for completeness and proper comparison of the results. Nevertheless, this investigation of the existing scientific literature highlighted the lack of a commonly accepted standard. This point will be addressed in a dedicated section.

For example, there has been much discussion on the influence that the storage of the samples could have on the mechanical properties of the specimens. The debate is still open, but it seems that freezing up to three months does not significantly modify the structural and mechanical properties of the samples, as proven by Woo and colleagues (Woo et al. 1986), studying the influence of conservation on rabbit MCL ligaments (Martin et al. 2015; Beynnon and Amis 1998). In fact, in the main part of the experimental studies reported in this review, the specimens were kept at low temperature (freezing) and defrosted shortly before the actual test. Generally, specimens were maintained hydrated in solution during tests. For the conservation of the specimens, a physiological solution is commonly used, but also the phosphate buffered saline and Ringer's solution are usable (Martin et al. 2015).

The aim of this study is to analyse the setup parameters used during the experimental tests. In particular, two main factors influence the mechanical response: (i) the strain rate and displacement rate values set during the test and (ii) the preconditioning before the test. These aspects will be discussed in detail in the rest of the paper.

### Difference between human and animals knee biomechanics

The substantial impact of knee ligaments injury, such as ACL, PCL, and collateral ligaments, has generated a big research field, thus allowing to explore their mechanisms of injury and the development of new treatment strategies. In fact, several large animal models are commonly used to study knee ligaments repair mechanisms, but no species is currently considered as the gold standard. However, each animal model has limitations, which should be carefully considered. Regarding the human ACL, it is well known that is anatomically divided into three bundles: the anteromedial (AM), intermediate (IM), and posterolateral (PL), each of them performing different functions within the knee joint. Other animal species as dog and goat ACL have only two bundles, rabbit ACL has not bundles, and only pig and goat ACL have three bundles (Bascuñán et al. 2019). Furthermore, biomechanical studies on the human ACL have shown that different bundles of ligaments have opposite behaviour during knee joint extension and flexion. Nevertheless, no animal ACL presents that mechanical behaviour in different portions (Bascuñán et al. 2019). Goat and swine appear to be a valid surrogate of ACL, since they present the greatest similarities with human ones (Bascuñán et al. 2019).

Another aspect to consider when experimental studies on knee animal models are designed is the difference in the mechanical properties of the knee ligaments at different angles of work. Wingfield et al. (2000) analysed the influence of two different knee angles in the mechanical properties of dog CraCl. However, no significant difference in the mechanical properties was found, but it is well known that cruciate ligaments in humans are influenced by the knee angle. Further studies need to evaluate more precisely this aspect.

## Materials and methods

### Eligibility criteria

The primary aim of this review is a systematic revision of the scientific literature reporting tensile-testing mechanical properties of healthy ligaments in different animal species (bovine, dog, equine, monkey, mouse, ovine, rabbit, rat, swine). The mechanical properties were collected to compare the mechanical behaviour and identify the most suitable animal model.

In the cases where the data were expressed in units of measures that did not belong to SI units, they were converted into the corresponding SI units. Furthermore, to improve data accuracy, the expression of these properties as mean value ± standard deviation (SD) was required. All articles that presented the following characteristics were excluded: (i) results of the tensile test represented only in a graphic form, expressed only as mean without standard deviation, percentage, or range of values; (ii) studies on pathological or damaged ligaments only; (iii) study conducted on ligaments harvested from paediatric or elderly patients; (iv) studies evaluating the healing process of injured ligaments through the insertion of allografts or autografts or that included the use of different kinds of scaffolds or growth factors; (v) studies that report only compression and shear stress values and viscoelastic properties of the specimens; (vi) studies with data derived from finite element models; (vii) studies that perform biaxial test.

### Information sources and search

The main databases were PubMed, Google Scholar, Science Direct, Springer, Taylor and Francis, Wiley-Blackwell, and PicoPolito (Politecnico di Torino search engine). The keywords used to find the articles in the primary research were: “ligaments”, “animal ligaments”, “human ligaments”, “biomechanics”, “mechanical characterisation”, “mechanical properties”, “structural properties”, “stress–strain”, “tensile test”, “failure test”, “strain rate”, “Young’s modulus”, “ultimate tensile stress”, and “ultimate strain”. All the collected data were exported to Microsoft Excel and analysed. The research was conducted by four authors (S.C., M.M., F.S., and G.S.) working independently, each of them investigating one-quarter of the number of articles analysed and then reviewing them together one by one over three months. This study was conducted according to the Preferred Reporting Items for Systematic Reviews and Meta-Analyses (PRISMA) method.

### Data items

Specifically, the following mechanical properties were considered: elastic modulus or Young’s modulus (MPa), stiffness ($N \, {\text{mm}}^{ - 1}$), maximal load (N), ultimate tensile stress (MPa), ultimate strain (%), and energy absorbed at failure ($N \, {\text{mm}}$). Additionally, regarding the experimental setup of the tensile tests, the preconditioning application, the strain rate ($\%\, {\text{min}}^{ - 1}$), and the displacement rate (${\text{mm\,min}}^{ - 1}$) values set for the tests were reported.

### Additional analysis

In order to evaluate all the aspects related to the experimental tensile tests, the two methodologies that are employed to perform the tests were considered: “strain-controlled mode” and “displacement-controlled mode”. The information about the control mode adopted by various authors during tensile tests was reported with the relative values of strain rate, where “SCM” stands for “strain controlled mode” and “DCM” stands for “displacement-controlled mode”.

Additionally, the type of preconditioning used for the tests was reported and evaluated to give some guidelines in the results section.

## Results

### Study selection

**Fig. 1:**
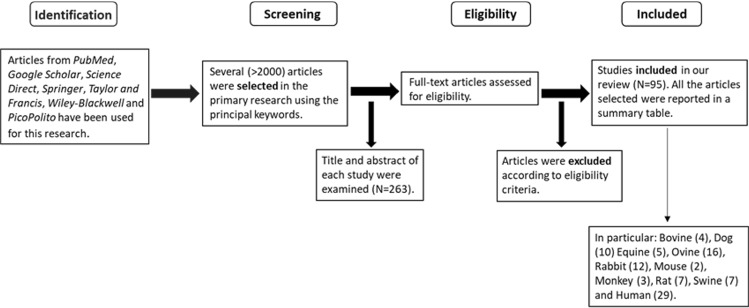
Workflow followed to identify, exclude and select the articles

The initial research of peer-reviewed articles published in the selected databases using the mentioned keywords includes more than 2000 manuscripts. Then, the title and abstracts were analysed to include the papers and 263 manuscripts for the full-text evaluation were selected. Following the eligibility criteria, 95 articles were evaluated to obtain values of the mechanical properties (Fig. 1).

In particular, data were classified in animal species as follows: cow (*n* = 3), calf (*n* = 1), dog (*n* = 10), horse (*n* = 5), foal (*n* = 1), monkey (*n* = 3), mouse (*n* = 2), goat (*n* = 6), sheep (*n* = 10), rabbit (*n* = 12), rat (*n* = 7), swine (*n* = 7), and human (*n* = 29). We considered any peer-reviewed article published in English between 1968 and the current date (May 2022).

### Synthesis of results

After selecting the articles that were in compliance with the eligibility criteria, all the data regarding ligament mechanical and the type of preconditioning used in the published studies were reported in many summary tables. Article summaries are illustrated in Table 1, grouped by animal species and human. Table 2 reports a list of the ligament acronyms as used in this paper.

**Table 1:** All the selected articles are grouped by animal species and human

Animal species	Studies
Bovine (Cow; Calf)	Niehaus et al. (2013), Diotalevi et al. (2018), Oskui et al. (2016); Eleswarapu et al. (2011)
Dog	Butler et al. (1983), Shino et al. (1984), Figgie et al. (1986), Nikolaou et al. (1986), Beynnon et al. (1994), Wingfield et al. (2000), Comerford et al. (2005), Dupuis et al. (1994), Shetye et al. (2009), Woo et al. (1990b)
Equine (Horse; Foal)	Riemersma and Schamhardt (1985), Jansen and Savelberg (1994), Smith (2006), Gellman and Bertram (2002), Becker et al. (1994)
Monkey	Noyes et al. (1974), Vardy et al. (2005), Noyes and Grood (1976)
Mouse	Carballo et al. (2018), El-Zawawy et al. (2005)
Ovine (Goat; Sheep)	McPherson et al. (1985), Jackson et al. (1988), Jackson et al. (1991), Jackson et al. (1993), Ng et al. (1995), Abramowitch et al. (2003); Rogers et al. (1990), Radford et al. (1996), Weiler et al. (2001), Weiler et al. (2004), Hunt et al. (2005), Meller et al. (2008), Gurlek et al. (2017), Viateau et al. (2013), Mahalingam et al. (2015), Mallett and Arruda (2017)
Rabbit	Woo et al. (1992), Danto and Woo (1993), Panjabi et al. (1996), Murao et al. (1997), Ma et al. (2009), Tozilli and Arnoczky (1988), Woo et al. (1986), Woo et al. (1990c), Woo et al. (1990a), Weiss et al. (1991), Moon et al. (2006), Xie et al. (2021)
Rat	Yiannakopoulos et al. (2005), Nawata et al. (2001), Belanger et al. (2000), Su et al. (2008), Lee et al. (2006), Freedman et al. (2012), Quinn and Winkelstein (2007)
Swine	Hirokawa and Sakoshita (2003), Zhou et al. (2009), Bonner et al. (2015), Germscheid et al. (2011), Kim et al. (2014), Polak et al. (2014), Tan et al. (2015)
Human	Trent et al. (1976), Noyes and Grood (1976), Chandrashekar et al. (2006), Woo et al. (1991), Race and Amis (1994), Sugita and Amis (2001), LaPrade et al. (2005), Ciccone et al. (2006), Wilson et al. (2012), Quapp and Weiss (1997), Wijdicks et al. (2010), Robinson et al. (2005), Zens et al. (2015), Criscenti et al. (2016), Kusayama et al. (1994), Gupte et al. (2002), Hewitt et al. (2002), Schleifenbaum et al. (2016), Pieroh et al. (2016), Neumann et al. (1994), Przybylski et al. (1996), Mattucci et al. (2012), Nachemson and Evans (1968), Lee et al. (1999), Bigliani et al. (1992), Moore et al. (2004), Moore et al. (2005), Fremerey et al. (2000), Johnston et al. (2004), (Martins et al. 2013)

**Table 2:** Acronyms list to indicate the ligaments quoted in this review

Part of the body	Type of ligament
Knee	Anterior cruciate ligament (ACL) [also called cranial cruciate ligament (CraCL) for the animals], posterior cruciate ligament (PCL) [also called caudal cruciate ligament (CauCL) for the animals], lateral collateral ligament (LCL) [also called fibular collateral ligament], medial collateral ligament (MCL), anterolateral ligament (AL), posterior oblique ligament (POL), medial patellofemoral ligament (MPFL), popliteofibular ligament (PFL), meniscofemoral ligament (MFL)
Hip joint	Iliofemoral ligament (IL), superior halves of the iliofemoral ligament (SHIL), inferior halves of the iliofemoral ligament (IHIL), ischiofemoral ligament (IS), Pubofemoral ligament (PF), femoral arcuate ligament (FAL)
Spinal cord	Facet capsular ligament (FCL), anterior longitudinal ligament (ALL), posterior longitudinal ligament (PLL), capsular ligament (CL), ligamentum flavum (LF), interspinous ligament (ISL), supraspinous ligament (SSL), nuchal ligament (NL), denticulate ligament (DL)
Shoulder	Anterior band of inferior glenohumeral ligament (AB-IGHL), Posterior band of inferior glenohumeral ligament (PB-IGHL), superior band of inferior glenohumeral ligament (SB-IGHL), inferior glenohumeral ligament (IGHL), coracoacromial ligament (CAL)
Limbs	Scapholunate ligament, accessorometacarpal ligament (AMCL), palmar radiocarpal ligament (PRL), palmar ulnocarpal ligament (PUL), accessory ligament (AccL), distal check ligament (DCL), suspensory ligament (SL)
Uterus	Uterosacral ligaments (USL), round ligaments (RL), cardinal ligament (CL)
Mouth	Periodontal ligament (PL)

### Study characteristics

Table 3 shows the mechanical properties (strain rate and/or displacement rate, Young’s modulus, stiffness, maximum load, ultimate tensile stress, ultimate strain, and energy absorbed at failure) in different animal species considering the control mode (only in the studies in which strain rate was used) and preconditioning. Table 4 reports the same mechanical values for different human ligaments.

**Table 3:** Mechanical properties of animal ligaments, grouped by species. ‘na’ indicates unavailable data

Type of ligament	Species/ breed	Population (*n*. of ligaments)	Preconditioning	Displacement rate [mm min^-1]^	Strain rate [% min^-1]^ MODE	Young’s Modulus [MPa]	Stiffness [${\text{N\,mm}}^{{ - 1}}$	Maximal load [N]	Ultimate tensile stress [MPa]	Ultimate strain [%]	Energy absorbed at failure [$N \, {\text{mm}}$	References
**BOVINE**												
**Cow**												
CraCL^a, b^	na	6	na	$60$	na	na	na	$4541 \pm 1417$	na	na	na	Niehaus et al. (2013)
CraCL	na	8	Yes	na	$120$ SCM	na	na	$4372 \pm 1485$	na	na	na	Diotalevi et al. (2018)
PL^a, c^	na	5	Yes	na	$60$ DCM	$5.85 \pm 0.04$	na	na	$2.65 \pm 0.14$	$170 \pm 4$	na	Oskui et al. (2016)
		5			$600$ DCM	$7.58 \pm 0.10$			$3.10 \pm 0.26$	$164 \pm 6$		
		5			$6000$ DCM	$10.64 \pm 0.27$			$3.40 \pm 0.31$	$156 \pm 3$		
		5			$60000$ DCM	$13.63 \pm 0.35$			$3.25 \pm 0.46$	$145 \pm 3$		
Calf												
CraCL	na	6	na	na	$60$ DCM	$2.1 \pm 1.0$	na	na	$1.4 \pm 0.6$	na	na	Eleswarapu et al. (2011)
CauCL	na	6	na	na	$60$ DCM	$11.6 \pm 5.9$	na	na	$7.4 \pm 5.9$	na	na	Eleswarapu et al. (2011)
LCL	na	6	na	na	$60$ DCM	$16.9 \pm 4.07$	na	na	$14.9 \pm 3.9$	na	na	Eleswarapu et al. (2011)
MCL	na	6	na	na	$60$ DCM	$13.2 \pm 5.8$	na	na	$10.1 \pm 6.4$	na	na	Eleswarapu et al. (2011)
**DOG**												
CraCL^a, b, d^	Mongrel	15	na	na	$6000$ DCM	$543.8 \pm 36.2$	$348.1 \pm 26.9$	$1656 \pm 125$	$146.7 \pm 9.2$	$36.4 \pm 2.5$	$(4.3 \pm 0.5)$ $\times 10^{3}$	Butler et al. (1983)
ACL^a, b, e^	Mongrel	5	na	$500$	na	na	na	$505.2 \pm 168.1$	na	$139.2 \pm 28.4$	$(2.23 \pm 0.70)$ $\times 10^{3}$	Shino et al. (1984)
		5				na	na	$454.2 \pm 45.3$	na	$170.6 \pm 62.7$	$(2.35 \pm 0.25)$ $\times 10^{3}$	
		4				na	na	$705.3 \pm 50.5$	na	$106.9 \pm 7.8$	$(3.23 \pm 0.57)$ $\times 10^{3}$	
		4				na	na	$637.9 \pm 69.7$	na	$94.1 \pm 10.8$	$(2.60 \pm 0.56)$ $\times 10^{3}$	
ACL^b ,f^	Beagle	9	na	$510$	na	na	na	$1181 \pm 276$	na	$91 \pm 6$	($9.76 \pm 2.23)$ $\times 10^{3}$	Figgie et al. (1986)
ACL^b, d^		9				na	na	$454 \pm 84$	na	$72 \pm 11$	$(3.25 \pm 0.64)$ $\times 10^{3}$	
ACL^b, c^		9				na	na	$428 \pm 77$	na	$51 \pm 11$	$(5.01 \pm 1.56)$ $\times 10^{3}$	
ACL^a, b, d^	Mongrel	20	na	na	$6000$ SCM	na	$8.58 \pm 0.17$	$115.1 \pm 9.3$	na	na	$(0.95 \pm 0.17)$ $\times 10^{3}$	Nikolaou et al. (1986)
		3				na	$9.22 \pm 0.21$	$183.9 \pm 24.2$	na	na	$(3.15 \pm 0.30)$ $\times 10^{3}$	
		3				na	$9.63 \pm 0.00$	$100.6 \pm 9.6$	na	na	$(1.29 \pm 0.23)$ $\times 10^{3}$	
		3				na	$8.82 \pm 0.49$	$96 \pm 11.7$	na	na	$(1.24 \pm 0.20)$ $\times 10^{3}$	
		3				na	$8.82 \pm 0.00$	$104.8 \pm 6.8$	na	na	$(1.38 \pm 0.22)$ $\times 10^{3}$	
		2				na	$8.89 \pm 0.32$	$129.4 \pm 3.9$	na	na	$(0.98 \pm 0.09)$ $\times 10^{3}$	
ACL^a, b, e^	na	12	na	$9$	$6000$ SCM	na	$201.5 \pm 41.2$	$1867 \pm 324$	na	na	na	Beynnon et al. (1994)
CraCL^b, g^	Rottweiler	7	na	$1000$	na	$198.7 \pm 35.95$	$303.3 \pm 20.88$	$1643 \pm 286.72$	$60.8 \pm 9.18$	$38.1 \pm 6.26$	$(5.6 \pm 1.51)$ $\times 10^{3}$	Wingfield et al. (2000)
CraCL^b, i^		6				$203.0 \pm 10.06$	$306.7 \pm 58.09$	$1738 \pm 475.89$	$63.3 \pm 8.91$	$35.1 \pm 1.73$	$(5.5 \pm 2.14)$ $\times 10^{3}$	
CraCL^b, h^	Racing Greyhound	5				$218.2 \pm 15.35$	$265.0 \pm 30.98$	$1421 \pm 150.25$	$72.0 \pm 4.36$	$37.7 \pm 3.33$	$(4.1 \pm 0.82)$ $\times 10^{3}$	
CraCL^b, i^		6				$221.0 \pm 17.78$	$263.0 \pm 17.31$	$1781 \pm 137.83$	$86.2 \pm 6.84$	$44.2 \pm 1.05$	$(5.8 \pm 0.73)$ $\times 10^{3}$	
ACL^b, e^	Greyhound	11	Yes	$1000$	na	$66.77 \pm 7.2$^**(s)**^	na	$820.0 \pm 32.8$	$40.16 \pm 3.5$	$50.3 \pm 4.7$	$(3.4 \pm 0.4)$ $\times 10^{3}$	Comerford et al. (2005)
	Labrador Retriever	11				$81.02 \pm 11.3$^**(s)**^	na	$704.4 \pm 45.6$	$31.0 \pm 3.1$	$45.9 \pm 2.7$	$(2.8 \pm 0.3)$ $\times 10^{3}$	
LCL^a, b, d^	na	5	na	$1000$	na	na	$81.0 \pm 18.4$	$570.2 \pm 214.6$	na	na	$(3.6 \pm 2.7)$ $\times 10^{3}$	Dupuis et al. (1994)
		3				na	$77.3 \pm 10.7$	$867.0 \pm 189.0$	na	na	$(5.1 \pm 1.9)$ $\times 10^{3}$	
		4				na	$87.7 \pm 8.1$	$832.3 \pm 118.3$	na	na	$(4.3 \pm 3.3)$ $\times 10^{3}$	
LCL^b^	na	8	Yes	na	$30$ SCM	na	$61.10 \pm 30.42$	$233.11 \pm 144.86$	na	na	na	Shetye et al. (2009)
MCL^a, b, k^	Mongrel	5	Yes	$20$	$30$ SCM	$620 \pm 60$	$54.3 \pm 2.7$	$664 \pm 65$	$72.3 \pm 4.8$	na	$4081 \pm 704$	Woo et al. (1990b)
		5				$670 \pm 130$	$65.0 \pm 4.1$	$606 \pm 68$	$81.4 \pm 26.6$	na	$2874 \pm 572$	
		5				$560 \pm 120$	$70.0 \pm 7.0$	$644 \pm 56$	$72.1 \pm 14.2$	na	$3060 \pm 360$	
		5				$470 \pm 100$	$53.2 \pm 5.0$	$760 \pm 54$	$79.5 \pm 16.9$	na	$4443 \pm 419$	
		5				$550 \pm 80$	$63.5 \pm 3.5$	$581 \pm 47$	$69.7 \pm 5.8$	na	$2650 \pm 360$	
		5				$660 \pm 230$	$53.9 \pm 2.3$	$768 \pm 144$	$84.6 \pm 18.1$	na	$4643 \pm 1329$	
MCL^b^	na	8	Yes	na	$30$ SCM	na	$72.65 \pm 10.86$	$392.45 \pm 132.61$	na	na	na	Shetye et al. (2009)
AMCL-IV^b^	na	8	Yes	na	$30$ SCM	$546.06 \pm 106.97$	$72.33 \pm 14.66$	$426.15 \pm 100.79$	na	na	na	Shetye et al. (2009)
AMCL-V^b^	na	8	Yes	na	$30$ SCM	$382.38 \pm 180.50$	$145.864 \pm 49.44$	$602.54 \pm 165.22$	na	na	na	Shetye et al. (2009)
PRL^b^	na	8	Yes	na	$30$ SCM	na	$80.20 \pm 41.21$	$149.27 \pm 68.61$	na	na	na	Shetye et al. (2009)
PUL^b^	na	8	Yes	na	$30$ SCM	na	$94.70 \pm 14.43$	$414.66 \pm 72.29$	na	na	na	Shetye et al. (2009)
**EQUINE**												
** Horse**												
SL	na	9	na	na	$60$ SCM	na	na	na	na	$11 \pm 0.6$	na	Riemersma and Schamhardt (1985)
SL-MT	na	6	na	na	$6$ SCM	$576 \pm 44$	na	na	na	na	na	
SL-MT	na	6	na	na	$60$ SCM	$588 \pm 47$	na	na	na	na	na	
SL-SS	na	6	na	na	$6$ SCM	$669 \pm 66$	na	na	na	na	na	
SL-SS	na	6	na	na	$60$ SCM	$683 \pm 67$	na	na	na	na	na	
SL	Horses and ponies	12	Yes	na	$60-180$ SCM	na	na	na	$70 \pm 12$	$11.6 \pm 1.4$	na	Jansen and Savelberg (1994)
SL	Irish, Thoroughbred Cross, 3 Thoroughbred, 7/8 Tb Irish Sport Horse/Eventing	6	Yes	na	$4800$ DCM	$643 \pm 130$	na	$\left(16.8 \pm 2.5\right)$ $\times {10}^{3}$	$91 \pm 19$	$20.2 \pm 5.2$	na	Smith (2006)
NL^j^	Grade gelding, Arab mare, Paint gelding Thoroughbred gelding, Standardbred gelding	18	na	$480$	na	$0.84 \pm 0.02$^**(t)**^	na	na	na	na	na	Gellman and Bertram (2002)
AccL^b^	Warmblood (young adult)	5	Yes	na	$60-180$ DCM	$990 \pm 427$	na	$4210 \pm 2823$	$85 \pm 29$	$13 \pm 4$	na	Becker et al. (1994)
	Warmblood (older)	6	Yes	na		$1002 \pm 209$	na	$8894 \pm 942$	$77 \pm 12$	$11 \pm 3$	na	
DCL	Horses and ponies	12	Yes	na	$60-180$ SCM	na	na	na	$86 \pm 11$	$12.4 \pm 1.0$	na	Jansen and Savelberg (1994)
**Foal**												
AccL^b^	Warmblood	10	Yes	na	$60-180$ DCM	$795 \pm 117$	na	$7835 \pm 2539$	$61 \pm 15$	$12 \pm 2$	na	Becker et al. (1994)
**MONKEY**												
ACL^b, d^	Rhesus monkey (Macaca mulatta)	17	na	$508.02$	na	na	na	$997.3 \pm 164.7$	na	$57.1 \pm 10.4$	$(4.12 \pm 1.09)$ $\times {10}^{3}$	Noyes et al. (1974)
ACL^b, d^	Rhesus monkey (Macaca mulatta)	17	na	$5.0802$	na	na	na	$805.1 \pm 175.5$	na	$51.9 \pm 10.1$	$(3.01 \pm 0.85)$ $\times {10}^{3}$	Noyes et al. (1974)
USL^a^	Macaca fascicularis	19	na	$6$	na	$0.08 \pm 0.05$	na	na	$0.6 \pm 0.4$	na	na	Vardy et al. (2005)
RL^a^	Macaca fascicularis	19	na	$6$	na	$3.19 \pm 2.62$	na	na	$2.1 \pm 1.1$	na	na	Vardy et al. (2005)
ACL^b, d^	Rhesus monkey	25	na	na	$3960$ SCM	$186 \pm 26$	$194 \pm 28$	$\left(0.83 \pm 0.11\right)$ $\times {10}^{3}$	$66.1 \pm 8.4$	na	($3.0 \pm 0.6)$ $\times {10}^{3}$	Noyes and Grood (1976)
MOUSE												
ACL^b, m^	Inbred wild-type (C57BL-6)	7	na	$10$	$100$ na	na	$3.44 \pm 1.47$	$5.60 \pm 0.75$	na	na	na	Carballo et al. (2018)
PCL^b, m^	Inbred wild-type (C57BL-6)	7	na	$10$	$100$ na	na	$3.99 \pm 0.98$	$3.33 \pm 1.45$	na	na	na	Carballo et al. (2018)
LCL^b, m^	Inbred wild-type (C57BL-6)	7	na	$10$	$100$ na	na	$1.35 \pm 0.87$	$1.44 \pm 0.37$	na	na	na	Carballo et al. (2018)
MCL^b, m^	Inbred wild-type (C57BL-6)	7	na	$10$	$100$ na	na	$3.02 \pm 1.08$	$3.45 \pm 3.84$	na	na	na	Carballo et al. (2018)
MCL^b, d^	129X1-SVJ (6 mo)	10 (right)	na	$15$	na	na	na	$11.6 \pm 2.7$	na	na	na	El-Zawawy et al. (2005)
		10 (left)	na	$15$	na	na	na	$7.2 \pm 2.6$	na	na	na	
**OVINE**												
**Goat**												
ACL ^a, b, e^	na	4	na	$150$	na	na	$275 \pm 23$	$2023 \pm 494$	na	na	na	McPherson et al. (1985)
ACL ^a, b, e^	Spanish	12	na	na	$6000$ DCM	na	$692 \pm 37$	$2403 \pm 133$	na	na	$(4.85 \pm 0.29)$ $\times 10^{3}$	Jackson et al. (1988)
ACL^a, b^	Spanish	6	na	na	$6000$ DCM	$517 \pm 40$	$448 \pm 38$	$2201 \pm 111$	$133 \pm 11$	$36 \pm 4$	na	Jackson et al. (1991)
		6				$629 \pm 26$	$524 \pm 22$	$2274 \pm 116$	$151 \pm 7$	$29 \pm 2$	na	
		6				$578 \pm 51$	$548 \pm 31$	$2603 \pm 213$	$155 \pm 13$	$32 \pm 2$	na	
ACL^a, b, e^	Spanish	24	na	na	$6000$ DCM	$310 \pm 37$	$352 \pm 21$	$2192 \pm 119$	$108.0 \pm 6.8$	na	na	Jackson et al. (1993)
ACL^a, b, c^	Mixed breed	4	Yes	na	$4800$ SCM	$380.2\pm 75.2$	$305.8\pm 67.4$	$1546.7\pm 464.4$	na	na	na	Ng et al. (1995)
MCL^a, b, k^	Saanen	6 (*5)	Yes	$10$	na	$516\pm 158$* ^**(u)**^	$72.7\pm 9.2$	$765\pm 149$	$62.0\pm 10.3$	na	$3951\pm 577$	Abramowitch et al. (2003)
**Sheep**												
ACL ^b, d^	na	9 (left)	na	$500$	na	$200\pm 30$	na	$2580\pm 320$	$122 \pm 12$	$71\pm 5$	na	Rogers et al. (1990)
		9 (right)	na	$500$	na	$210\pm 20$	na	$2570\pm 470$	$124\pm 19$	$68\pm 6$		
ACL ^b^	na	4 (left)	na	$500$	na	$240\pm 30$	na	$1900\pm 160$	$81\pm 12$	$45\pm 2$	na	
		4 (right)	na	$500$	na	$240 \pm 50$	na	$1920 \pm 120$	$80 \pm 11$	$46 \pm 6$		
CraCL^b^	Pentland cross-bred	18	na	$100$	na	na	na	$2354 \pm 235$	na	na	na	Radford et al. (1996)
ACL^a, b^	Merino	12	na	$60$	na	na	$143.9 \pm 16.1$	$1531.3 \pm 180.3$	$53.6 \pm 13.6$	na	na	Weiler et al. (2001)
ACL^a, b^	na	16	Yes	$60$	na	na	$104.2 \pm 15.2$	$888 \pm 139$	$41.9 \pm 4.5$	na	na	Weiler et al. (2004)
ACL ^a, b, c^	Merino	12	na	$60$	na	na	$143.9 \pm 16.1$	$1513.3 \pm 180.3$	$53.6 \pm 13.6$	na	na	Hunt et al. (2005)
ACL ^a, b, c^	German moorland	10	na	$60$	na	na	$104.2 \pm 15.2$	$888.2 \pm 139.4$	$41.9 \pm 4.5$	na	na	Hunt et al. (2005)
ACL^a, b^	Black- headed	32	na	$6$	na	$217.3 \pm 57.9$	$136.3 \pm 28.5$	$759.2 \pm 114.1$	na	na	na	Meller et al. (2008)
ACL^a, b^	na	na	na	$5$	na	$2.288 \pm 0.568$	$144.97 \pm 35.34$	$548.78 \pm 41.44$	$35.71 \pm 2.34$	$46.48 \pm 14.09$	na	Gurlek et al. (2017)
ACL^a, b^	Pré-Alpes	7	Yes	$5$	na	na	na	$1241 \pm 270$	na	na	na	Viateau et al. (2013)
		8		$5$		na	na	$1218 \pm 189$	na	na	na	
ACL^a, b, e^	Black Suffolk	12	na	na	$300$ DCM	$158 \pm 32$^**(ag)**^	na	na	na	na	na	Mahalingam et al. (2015)
Antero- medial ACL ^b^	Suffolk	20	na	na	$300$ DCM	$\left(1.07 \pm 0.17\right)$ $\times 10^{3}$^**(ah)**^	na	na	na	na	na	Mallett and Arruda (2017)
		20	na			$(0.37 \pm 0.17)$ $\times 10^{3}$^**(ai)**^	na	na	na	na	na	
		20	na			$\left(1.04 \pm 0.24 \right)$ $\times 10^{3}$^**(aj)**^	na	na	na	na	na	
		20	na			$(0.33 \pm 0.19)$ $\times 10^{3}$^**(ak)**^	na	na	na	na	na	
**RABBIT**												
Medial ACL^b^	New Zealand	10	Yes	$10$	na	$516 \pm 64$^**(u)**^	na	na	na	na	na	Woo et al. (1992)
Lateral ACL^b^		10				$516 \pm 69$^**(u)**^	na	na	na	na	na	
Medial ACL^b, c, l^	New Zealand	18	Yes	na	$0.96 \pm 0.06$ DCM	$711 \pm 18$^**(v)**^	na	na	na	na	na	Danto and Woo (1993)
				na	$100.8 \pm 9.6$ DCM	$674 \pm 62$^**(v)**^	na	na	na	na	na	
				na	$22860 \pm 2160$ DCM	$930 \pm 71$^**(v)**^	na	na	na	na	na	
ACL^a, b^	New Zealand	13	Yes	$60$	$\sim 600$ $\mathrm{DCM}$	na	na	$164.6 \pm 38.5$	na	na	$192.8 \pm 62.3$	Panjabi et al. (1996)
PCL^a, b, d^	Japanese (male, 3 mo)	7	Yes	200	na	na	$229 \pm 57$	$388 \pm 76$	na	na	$502 \pm 95$	Murao et al. (1997)
	Japanese (male, 6 mo)	7				na	$263 \pm 27$	$457 \pm 86$	na	na	$584 \pm 252$	
PCL^a, b, d, k^	New Zealand	6	Yes	200	na	na	$106 \pm 9.32$	$169.27 \pm 12.71$	na	na	$226.20 \pm 20.69$	Ma et al. (2009)
		6				na	$116.39 \pm 9.53$	$187.41 \pm 13.99$	na	na	$284.28 \pm 40.41$	
LCL^a b k^	New Zealand	5	Yes	$6120$	na	na	na	na	na	na	$282 \pm 132$	Tozilli and Arnoczky (1988)
MCL^a, b, c, j^	New Zealand	5	Yes	$10$	$24$ SCM	na	na	$368.4 \pm 15.0$	na	na	$1330.0 \pm 200.0$	Woo et al. (1986)
MCL^b, c^	New Zealand (male, 3.5 mo)	6	na	$10$	na	$700 \pm 50$^**(z)**^	$40.0 \pm 1.7$	$88.4 \pm 7.8$	na	na	$(0.12 \pm 0.02)$ $\times 10^{3}$	Woo et al. (1990c)
	New Zealand (male, 6 mo)	6	na			$630 \pm 110$^**(z)**^	$45.0 \pm 4.5$	$156.5 \pm 22.9$	$46.0 \pm 0.7$	$11.2 \pm 2.1$	$(0.29 \pm 0.08)$ $\times 10^{3}$	
	New Zealand (male, 12 mo)	6	na			$1180 \pm 90$^**(z)**^	$64.2 \pm 5.7$	$313.2 \pm 18.8$	$84.4 \pm 5.7$	$10.6 \pm 0.8$	$(0.86 \pm 0.07)$ $\times 10^{3}$	
	New Zealand (male, 36 mo)	6	na			$740 \pm 90$^**(z)**^	$50.6 \pm 3.0$	$299.8 \pm 8.6$	$77.7 \pm 1.9$	$12.9 \pm 1.2$	$(1.05 \pm 0.14)$ $\times 10^{3}$	
	New Zealand (female, 3.5 mo)	6	na			$750 \pm 70$^**(z)**^	$31.6 \pm 2.0$	$87.8 \pm 4.4$	na	na	$(0.13 \pm 0.02)$ $\times 10^{3}$	
	New Zealand (female, 6 mo)	6	na			$590 \pm 90$^**(z)**^	$34.0 \pm 2.8$	$117.7 \pm 12.9$	na	na	$(0.23 \pm 0.04)$ $\times 10^{3}$	
	New Zealand (female, 12 mo)	14	na			$950 \pm 80$^**(z)**^	$48.2 \pm 3.1$	$290.4 \pm 20.2$	$75.8 \pm 4.8$	$9.4 \pm 0.7$	$(0.77 \pm 0.15)$ $\times 10^{3}$	
	New Zealand (female, 36 mo)	6	na			$710 \pm 30$^**(z)**^	$51.8 \pm 4.2$	$311.6 \pm 26.4$	$78.6 \pm 3.2$	$13.3 \pm 0.5$	$(0.98 \pm 0.16)$ $\times 10^{3}$	
	New Zealand (female, 3.5 mo)	6	na			$520 \pm 120$^**(z)**^	$50.3 \pm 2.9$	$267.0 \pm 26.7$	$68.9 \pm 4.9$	$11.9 \pm 1.3$	$(0.77 \pm 0.15)$ $\times 10^{3}$	
MCL^b, c^	New Zealand (male, 3.5 mo)	6	Yes	na	$0.66 \pm 0.06$ DCM	$610 \pm 100$^**(z)**^	$24.4 \pm 1.2$	$54.3 \pm 3.0$	na	na	$(0.07 \pm 0.01)$ $\times 10^{3}$	Woo et al. (1990a)
					$9 \pm 0.6$ DCM	$620 \pm 120$^**(z)**^	$40.0 \pm 2.4$	$84.5 \pm 8.5$	na	na	$(0.09 \pm 0.01)$ $\times 10^{3}$	
					$95.4 \pm 5.4$ DCM	$700 \pm 60$^**(z)**^	$40.0 \pm 1.9$	$88.4 \pm 8.6$	na	na	$(0.12 \pm 0.02)$ $\times 10^{3}$	
					$720 \pm 60$ DCM	$860 \pm 70$^**(z)**^	$41.2 \pm 3.3$	$105.7 \pm 8.9$	na	na	$(0.16 \pm 0.02)$ $\times 10^{3}$	
					$9300 \pm 11.40$ DCM	$970 \pm 190$^**(z)**^	$38.1 \pm 1.4$	$123.7 \pm 6.7$	na	na	$(0.20 \pm 0.02)$ $\times 10^{3}$	
	New Zealand (male, 8.5 mo)	6	Yes	na	$0.66 \pm 0.06$ DCM	$700 \pm 70$^**(z)**^	$52.8 \pm 1.6$	$311.5 \pm 12.1$	$75.2 \pm 2.4$	$9.5 \pm 0.5$	$(0.98 \pm 0.08)$ $\times 10^{3}$	
					$9 \pm 0.6$ DCM	$840 \pm 150$^**(z)**^	$54.8 \pm 1.4$	$295.8 \pm 28.1$	$81.4 \pm 7.4$	$10.0 \pm 0.5$	$(0.83 \pm 0.14)$ $\times 10^{3}$	
					$99.6 \pm 6.6$ DCM	$800 \pm 60$^**(z)**^	$54.3 \pm 2.5$	$337.5 \pm 23.7$	$85.7 \pm 7.7$	$11.0 \pm 0.5$	$(1.10 \pm 0.11)$ $\times 10^{3}$	
					$1116 \pm 150$ DCM	$910 \pm 50$^**(z)**^	$59.6 \pm 2.8$	$347.1 \pm 13.7$	$87.2 \pm 6.0$	$11.5 \pm 1.0$	$(1.10 \pm 0.09)$ $\times 10^{3}$	
					$13320 \pm 1500$ $\mathrm{DCM}$	$760 \pm 160$^**(z)**^	$60.7 \pm 1.7$	$403.7 \pm 7.5$	$106.7 \pm 6.8$	$13.0 \pm 1.0$	$\left(1.35 \pm 0.06\right)$ $\times 10^{3}$	
MCL^a, b, k^	New Zealand	6	Yes	10	na	$726 \pm 47$^**(aa)**^	$52.5 \pm 3.9$	$308 \pm 9$	$87.6 \pm 4.4$	$13.7 \pm 0.7$	$1031 \pm 115$	Weiss et al. (1991)
		6				$761 \pm 33$^**(aa)**^	$58.5 \pm 3.3$	$319 \pm 21$	$84.4 \pm 5.8$	$13.7 \pm 0.5$	$936 \pm 91$	
		6				$824 \pm 54$^**(aa)**^	$54.3 \pm 4.4$	$294 \pm 11$	$92.8 \pm 5.4$	$14.6 \pm 0.5$	$793 \pm 33$	
		6				$1080 \pm 101$^**(aa)**^	$64.2 \pm 5.2$	$313 \pm 17$	$84.4 \pm 5.2$	$10.6 \pm 0.8$	$862 \pm 64$	
MCL^b, c^	New Zealand	10	Yes	$10$	na	$1120 \pm 153$^**(u)**^	na	na	$110 \pm 19$	$14.5 \pm 1.0$	na	Woo et al. (1992)
MCL^a, b j^	New Zealand	6	Yes	$10$	$$\begin{array}{c}27 \pm 4.8\\ SCM\end{array}$$	$1107.2 \pm 126.3$^**(aa)**^	$106.5 \pm 5.9$	$331.0 \pm 81.8$	$84.4 \pm 22.2$	$10.6 \pm 2.8$	$703.8 \pm 270.1$	Moon et al. (2006)
MCL^a, b, k^	na	6	Yes	5	na	$3116 \pm 267$^**(ab)**^	na	$56.79 \pm 3.22$	na	na	na	Xie et al. (2021)
		6				$3213 \pm 296$^**(ab)**^	na	$62.24 \pm 4.69$	na	na	na	
		6				$3258 \pm 291$^**(ab)**^	na	$66.22 \pm 3.45$	na	na	na	
		6				$3288 \pm 323$^**(ab)**^	na	$66.22 \pm 3.45$	na	na	na	
**RAT**												
ACL^b, n^	Wistar	30 (left)	na	$150$	na	na	$14.4 \pm 5.1$	$34.6 \pm 4.2$	na	na	$\left(6.1 \pm 2.5\right)$ $\times 10^{-2}$	Yiannakopoulos et al. (2005)
	Wistar	30 (right)	na	$150$	na	na	$16.3 \pm 3.5$	$32.4 \pm 5.1$	na	na	$\left(6.3 \pm 3.5\right)$ $\times 10^{-2}$	
ACL^a, b, o^	Sprague–Dawley	10	na	na	$600$ DCM	na	$41.3 \pm 19.3$	$26.7 \pm 8.6$	na	na	$\left(4.0 \pm 3.8\right)$ $\times 10^{-2}$	Nawata et al. (2001)
ACL^a, b, c^	Sprague–Dawley (female)	12	na	$30$	na	na	$14.8 \pm 5.2$	$41.2 \pm 5.6$	na	na	$80.1 \pm 24.8$	Belanger et al. (2000)
	Sprague–Dawley (male)	12	na	$30$	na	na	$15.3 \pm 3.3$	$44.3 \pm 9.4$	na	na	$74.7 \pm 27.3$	
MCL^b^	Wistar	30 (left)	na	100	na	na	$19.5 \pm 3.2$	$29.3 \pm 4.7$	na	na	$18.2 \pm 3.1$	Yiannakopoulos et al. (2005)
		30 (right)	na		na	na	$20.6 \pm 2.5$	$31.4 \pm 5.3$	na	na	$18.6 \pm 4.3$	
MCL^a, b, p, q^	Sprague–Dawley	10	Yes	$30$	na	$279 \pm 45$	$15.75 \pm 3.2$	$19.5 \pm 4.1$	$41.8 \pm 8.9$	$19.2 \pm 2.8$	$24 \pm 10$	Su et al. (2008)
MCL^a, b, p, r^						$314 \pm 43$	$17.5 \pm 2.1$	$22.6 \pm 3.6$	$51.3 \pm 10.8$	$22.0 \pm 3.4$	$32 \pm 8$	
FCL^b^	Holtzman	11	na	$4.8$	na	na	$0.75 \pm 0.27$	$2.96 \pm 0.69$	na	$41.3 \pm 20.0$	$8 \pm 5$	Lee et al. (2006)
Thoracic FCL (T3/T4)^a^	na	4	Yes	$4.8$	na	na	$2.32 \pm 0.66$	$1.70 \pm 0.48$	na	na	na	Freedman et al. (2012)
Cervical FCL (C6/C7)^b^	Holtzman	8	Yes	$4.8$	na	na	$5.45 \pm 1.07$	$2.45 \pm 0.60$	na	$151 \pm 111$	$1.05 \pm 0.44$	Quinn and Winkelstein (2007)
SWINE												
ACL	na	5	na	$19.8$	na	$104.2 \pm 20.4$^**(ac)**^	na	na	$21.5 \pm 3.6$	$30.7 \pm 5.1$	na	Hirokawa and Sakoshita (2003)
ACL^b^	na	4	na	$19.8$	na	$204.5 \pm 29.9$^**(ae)**^	na	na	$25.4 \pm 5.3$	$16.9 \pm 4.7$	na	
ACL^b^	na	11	na	$19.8$	na	$147.76 \pm 61.75$	$147.02 \pm 29.79$	$853.53 \pm 252.32$	$32.22 \pm 15.63$	$32 \pm 8$	$3715.36 \pm 1991.53$	Zhou et al. (2009)
Antero- Medial ACL^b^	na	11	na	$19.8$	na	$111.12 \pm 30.09$	$79.17 \pm 15.73$	$453.47 \pm 127.61$	$19.65 \pm 6.91$	$27 \pm 6$	$1739.19 \pm 533.02$	
Postero- Lateral ACL^b^	na	8	na	$19.8$	na	$123.32 \pm 45.55$	$130.27 \pm 27.47$	$588.81 \pm 144.55$	$23.27 \pm 6.66$	$30 \pm 10$	$2057.88 \pm 1110.51$	
PCL	na	6	na	$19.8$	na	$85.6 \pm 11.6$^**(ac)**^	na	na	$19.7 \pm 3.2$	$32.4 \pm 4.9$	na	Hirokawa and Sakoshita (2003)
PCL^b^	na	3	na	$19.8$	na	$312.8 \pm 35.0$^**(ad)**^	na	na	$33.8 \pm 4.2$	$11.2 \pm 2.5$	na	
LCL^b^	Large-white	7	Yes	na	$60$ SCM	$288 \pm 83.9$	na	na	$39.9 \pm 11.0$	$17 \pm 3$	na	Bonner et al. (2015)
		7		na	$600$ SCM	$364 \pm 86.6$	na	na	$56.5 \pm 8.2$	$18 \pm 3$	na	
		7		na	$5640$ SCM	$656 \pm 82.4$	na	na	$72.8 \pm 11.1$	$14 \pm 2$	na	
		7		na	$63600$ SCM	$763 \pm 141.3$	na	na	$75.9 \pm 9.6$	$11 \pm 3$	na	
		12		na	$779400$ SCM	$906 \pm 195.6$	na	na	$77.4 \pm 15.4$	$9 \pm 2$	na	
MCL^b, o^	Yorkshire	6 (right)	Yes	20	na	$400.0 \pm 47.5$^**(af)**^	$81.35 \pm 9.95$	$941.80 \pm 61.50$	$71.5 \pm 15.6$	$21.0 \pm 3.0$	na	Germscheid et al. (2011)
		6 (left)	Yes			$477.8 \pm 110.7$^**(af)**^	$83.54 \pm 9.6$	$999.08 \pm 135.68$	$88.5 \pm 19.1$	$22.3 \pm 2.8$	na	
MCL^b, o^	Red Duroc	6 (right)	Yes	20	na	$327.6 \pm 54.4$^**(af)**^	$82.92 \pm 8.03$	$924.95 \pm 55.93$	$53.4 \pm 7.4$	$19.5 \pm 2.4$	na	
		6 (left)	Yes			$390.0 \pm 109.9$^**(af)**^	$79.64 \pm 12.47$	$33.28 \pm 86.34$	$56.6 \pm 12.5$	$18.2 \pm 3.0$	na	
MPFL^b^	Yorkshire	11 (natural orientation)	Yes	10	na	na	$65 \pm 13$	$438 \pm 128$	na	na	$2141 \pm 927$	Kim et al. (2014)
		11 (non-natural orientation)	Yes			na	$50 \pm 17$	$386 \pm 136$	na	na	$1828 \pm 1078$	
DL	Domestic	67	na	$2$	na	na	na	$0.88 \pm 0.19$	$1.95 \pm 0.76$	$117 \pm 46$	na	Polak et al. (2014)
CL	na	7 (left)	Yes	45	na	$3.449 \pm 1.449$^**(al)**^	na	na	$0.854 \pm 0.207$	$42.4 \pm 13.9$	na	Tan et al. (2015)
		6 (right)			na	$5.385 \pm 2.424$^**(al)**^	na	na	$1.278 \pm 0.499$	$33.7 \pm 16.6$	na	
USL	na	5	Yes	$45$	na	$29.816 \pm 7.378$^**(al)**^	na	na	$2.767 \pm 0.444$	$21.6 \pm 5.8$	na	

^a^Values referred to the control group; ^b^ Values referred to the bone–ligament–bone complex; ^c^ Values referred to a knee flexion of 90°; ^d^ Values referred to a knee flexion of 45°; ^e^ Values referred to a knee flexion of 30°; ^f^ Values referred to a knee flexion of 0°; ^g^ Values referred to a knee flexion of 160°; ^h^ Values referred to a knee flexion of 150°; ^i^ Values referred to a knee flexion of 130°; ^j^ Values referred to the fresh samples group; ^k^ Values referred to sham operation; ^l^ Values referred to a knee flexion of 50°; ^m^ Values referred to a knee flexion of 35°; ^n^ Values referred to a knee flexion of 60°; ^o^ Values referred to a flexion of 80°; ^p^ Values referred to a flexion of 70°; ^q^ Values referred to non-cyclic group; ^r^ Values referred to cyclic group; ^s^ Tangent modulus at 200 N; ^t^ Young’s modulus found for the range of strain 0–0.50% and averaged; ^u^ Young’s modulus calculated between 4 and 7% strain of the stress–strain curve; ^v^ Young’s modulus measured as the slope of the stress–strain curve between 4.0 and 6.5% strain; ^z^ Young’s modulus measured as the slope of the stress–strain curve between 2 and 4% strain; ^aa^ Young’s modulus measured as the slope of the stress–strain curve between 3 and 5% strain; ^ab^ Young’s modulus obtained from the unloading stage; ^ac^ Young’s modulus calculated at 10–20% strain of the stress–strain curve; ^ad^ Young’s modulus calculated at 2–6% strain of the stress–strain curve; ^ae^ Young’s modulus calculated at 4–8% strain of the stress–strain curve; ^af^ Young’s modulus is the slope of the linear regression between 15 and 65% of the failure stress on the failure stress–strain curve, ^ag^ Young’s modulus at a strain range of 0.04–0.10; ah Young’s modulus referred to DIC, at 20 MPa stress; ^ai^ Young’s modulus referred to grip-to-grip at 20 MPa stress; ^aj^ Young’s modulus referred to DIC, at 3% strain; ^ak^ Young’s modulus referred to grip-to-grip at 3% strain; ^al^ Young’s modulus calculated by considering only the stress–strain data in the interval (: strain at the Ultimate Tensile Strength (UTS))

**Table 4:** Mechanical properties of human ligaments. ‘na’ indicates unavailable data

Type of ligament	Population (n. of ligaments)	Preconditioning	Displacement rate ${\text{mm}} \, {\min}^{{ - 1}}$	Strain rate $\% \, {\min}^{{ - 1}}$ MODE	Young’s Modulus [MPa]	Stiffness [${\text{N}} \, {\text{mm}}^{{ - 1}}$	Maximal load [N]	Ultimate tensile stress [MPa]	Ultimate strain [%]	Energy absorbed at failure [${\text{N}} \, {\text{mm}}$	Reference
Human											
ACL ^b, e^	6	Yes	50	na	na	$138.4 \pm 96.7$	$621.1 \pm 545.1$	na	na	na	Trent et al. (1976)
PCL ^b, e^	6				na	$179.8 \pm 63.7$	$739.6 \pm 403.3$	na	na	na	
LCL ^b, e^	5				na	$59.8 \pm 41.7$	$376.6 \pm 191.4$	na	na	na	
MCL ^b, e^	4				na	$70.6 \pm 16.2$	$516.1 \pm 222.2$	na	na	na	
ACL ^a, b^	20	na	na	$6000$ SCM	$65.3 \pm 24.0$	$129 \pm 39$	$\left(0.734 \pm 0.266\right)$ $\times {10}^{3}$	$13.3 \pm 5.0$	na	$(4.89 \pm 2.36)$ $\times {10}^{3}$	Noyes and Grood (1976)
ACL ^e, g^	10 (female)	Yes	na	$6000$ DCM	$99 \pm 50$	$199 \pm 88$	$1266 \pm 527$	$22.58 \pm 8.92$	$27 \pm 8$	$4691 \pm 3623$	Chandrashekar et al. (2006)
	10 (male)	Yes	na	$6000$ DCM	$128 \pm 35$	$308 \pm 89$	$1818 \pm 699$	$26.35 \pm 10.08$	$30 \pm 6$	$7280 \pm 3624$	
ACL ^a, c^	8	Yes	$20$	na	na	$220 \pm 24$	$1503 \pm 83$	na	na	$(6.1 \pm 0.5)$ $\times {10}^{3}$	Woo et al. (1991)
Antero-lateral PCL^e, k^	10	na	$1000$	$3000$ DCM	$248 \pm 119$	$347 \pm 140$	$1620 \pm 500$	$35.9 \pm 15.2$	$18.0 \pm 5.3$	na	Race and Amis (1994)
Postero-medial PCL^e, i^	10	na			$145 \pm 69$	$77 \pm 32$	$258 \pm 83$	$24.4 \pm 10.0$	$19.5 \pm 5.4$	na	
LCL^c, e, j^	10	No	$200$		na	$58.1 \pm 22.8$	$309 \pm 91$	na	$16.1 \pm 2.5$	na	Sugita and Amis (2001)
FCL^d, e^	8	Yes		$>6000$ DCM	$183.5 \pm 110.7$	$33.5 \pm 13.4$	$295 \pm 96$	$26.9 \pm 11.7$	$0.16 \pm 0.05$	na	LaPrade et al. (2005)
LCL^a, d, e^	13	Yes		$6000$ DCM	na	$82 \pm 25$	$460 \pm 163$	na	na	na	Ciccone et al. (2006)
LCL^d, e^	9	Yes	$50$	$\sim 1200$ DCM	na	$59 \pm 12$	$392 \pm 104$	na	na	$(2 \pm 1)$ $\times {10}^{3}$	Wilson et al. (2012)
MCL^d, m^	9	Yes	na	$60$ SCM	$332.15 \pm 58.27$	na	na	$38.56 \pm 4.76$	$17.11 \pm 1.53$	na	Quapp and Weiss (1997)
MCL^d, n^	7				$11.02 \pm 3.57$	na	na	$1.69 \pm 0.53$	$11.7 \pm 0.93$	na	
Distal Superficial MCL^d, e^	8	Yes	$20$	na	na	$63.1 \pm 9.1$	$557.1 \pm 55.4$	na	na	na	Wijdicks et al. (2010)
Proximal Superficial MCL^d, e^	8	Yes	$20$	na	na	$17.6 \pm 10.7$	$87.6 \pm 36.1$	na	na	na	
Deep MCL^d, e^	8	Yes	$20$	na	na	$27.6 \pm 5.0$	$100.5 \pm 10.3$	na	na	na	
Superficial MCL	8	Yes	$1000$	na	na	$80 \pm 8$	$534 \pm 85$	na	na	na	Robinson et al. (2005)
Deep MCL	8	Yes	$1000$	na	na	$42 \pm 14$	$194 \pm 82$	na	na	na	
MCL^d, )^	9	Yes	$50$	$\sim 1200$ DCM	na	$63 \pm 14$	$799 \pm 209$	na	na	$(6 \pm 3)$ $\times {10}^{3}$	Wilson et al. (2012)
AL^d, e^	4	na	$30$	na	$1.20 \pm 0.44$^**(o)**^	na	$49.90 \pm 14.62$	$32.78 \pm 4.04$	$35.96 \pm 4.47$	na	Zens et al. (2015)
POL^d, e^	8	Yes	$20$	na	na	$38.6 \pm 16.0$	$256.2 \pm 29.5$	na	na	na	Wijdicks et al. (2010)
MPFL^a, e, m^	24	Yes	na	$18$ SCM	$116 \pm 95$^**(p)**^	na	na	$16 \pm 11$	$24.3 \pm 6.8$	na	Criscenti et al. (2016)
PFL^c, e, l^	10	na	$200$	na	na	$43.6 \pm 14.8$	$186 \pm 65$	na	$17.0 \pm 5.2$	na	Sugita and Amis (2001)
PFL^d, e^	8	Yes	$>6000$	na	$24.8 \pm 14.5$	$28.6 \pm 13.6$	$298.5 \pm 144.1$	$12.8 \pm 6.0$	$0.64 \pm 0.40$	na	LaPrade et al. (2005)
MFL^d, e^	26	Yes	$200$	na	$355.1 \pm 234.0$^**(q)**^	$49.0 \pm 18.4$	$297.4 \pm 141.4$	na	na	$1125.4 \pm 735.8$	Kusayama et al. (1994)
Anterior MFL^d, e^	12	Yes	$200$	na	$281.3 \pm 239.6$^**(r)**^	na	$300.5 \pm 155.0$	na	na	na	Gupte et al. (2002)
Posterior MFL^d, e^	11				$226.9 \pm 127.5$^**(r)**^	na	$302.5 \pm 157.9$	na	na	na	
SHIL^d, e^	10	Yes	$2.4$	na	na	$97.8 \pm 67.5$	$320.3 \pm 267.7$	$2.90 \pm 1.52$	$7.7 \pm 2.2$	$(0.95 \pm 1.07)$ $\times {10}^{3}$	Hewitt et al. (2002)
IHIL^d, e^	10	Yes	$2.4$	na	na	$100.7 \pm 54.0$	$351.3 \pm 159.4$	na	$10.3 \pm 5.0$	$(1.17 \pm 0.76)$ $\times {10}^{3}$	
IL^b^	40	Yes	$20$	na	$24.4 \pm 21.0$	na	na	$10.0 \pm 7.6$	$84.5 \pm 36.0$	na	Schleifenbaum et al. (2016)
IL	18	na	$5$	na	$48.8 \pm 21.4$	na	na	na	$129.8 \pm 11.1$	na	Pieroh et al. (2016)
IS^d, e^	10	Yes	$2.4$	na	na	$36.9 \pm 24.4$	$136.0 \pm 74.6$	$2.29 \pm 1.69$	$2.29 \pm 1.69$	$(0.44 \pm 0.36)$ $\times {10}^{3}$	Hewitt et al. (2002)
IS^b^	40	Yes	$20$	na	$22.42 \pm 21.1$	na	na	$7.7 \pm 6.9$	$86.1 \pm 30.0$	na	Schleifenbaum et al. (2016)
IS	9	na	$5$	na	$37.5 \pm 20.4$	na	na	na	$128.7 \pm 13.7$	na	Pieroh et al. (2016)
PF^b^	40	Yes	$20$	na	$24.9 \pm 30.8$	na	na	$6.5 \pm 4.2$	$72.43 \pm 33.21$	na	Schleifenbaum et al. (2016)
PF	17	na	$5$	na	$49.0 \pm 32.1$	na	na	na	$133.2 \pm 23.7$	na	Pieroh et al. (2016)
FAL^d, e^	10	Yes	$2.4$	na	na	$10.4 \pm 4.4$	$78.2 \pm 37.9$	$6.61 \pm 3.52$	$15.0 \pm 7.5$	$(0.43 \pm 0.33)$ $\times {10}^{3}$	Hewitt et al. (2002)
ALL^e^	15	na	$6$	na	na	$81.7 \pm 37.2$	$742 \pm 384$	na	na	$(5.23 \pm 3.73)$ $\times {10}^{3}$	Neumann et al. (1994)
	24	na	$60-240$	na	na	$85.2 \pm 32.6$	$843 \pm 356$	na	na	$(6.60 \pm 4.54)$ $\times {10}^{3}$	
	15	na	$1020-13800$	na	na	$200 \pm 99.6$	$1261 \pm 369$	na	na	$(8.25 \pm 8.28)$ $\times {10}^{3}$	
ALL (C6/C7) ^d, e^	20	na	$19.8$	na	$48 \pm 50$	$48 \pm 19$	$105 \pm 44$	na	na	$154 \pm 83$ $N\, \mathrm{mm}$	Przybylski et al. (1996)
ALL (C5/C6) ^d, e^	20	na	$19.8$	na	$51 \pm 13$	$57 \pm 30$	$104 \pm 54$	na	na	$135 \pm 92$ $N\, \mathrm{mm}$	
ALL (C4/C5) ^d, e^	20	na	$19.8$	na	$42 \pm 22$	$54 \pm 27$	$106 \pm 61$	na	na	$168 \pm 198$ $N\, \mathrm{mm}$	
ALL (C3/C4) ^d, e^	20	na	$19.8$	na	$13 \pm 8$	$37 \pm 31$	$104 \pm 99$	na	na	$174 \pm 160$ $N\, \mathrm{mm}$	
ALL (C2/C3) ^d, e^	20	na	$19.8$	na	$75 \pm 48$	$43 \pm 29$	$66 \pm 37$	na	na	$70 \pm 33$ $N\, \mathrm{mm}$	
PLL (C6/C7) ^c, e^	20	na	$19.8$	na	$21 \pm 14$	$55 \pm 37$	$95 \pm 65$	na	na	$141 \pm 97$ $N\, \mathrm{mm}$	Przybylski et al. (1996)
PLL (C5/C6) ^c, e^	20	na	$19.8$	na	$22 \pm 10$	$65 \pm 33$	$89 \pm 42$	na	na	$84 \pm 42$ $N\, \mathrm{mm}$	
PLL (C4/C5) ^c, e^	20	na	$19.8$	na	$48 \pm 66$	$90 \pm 84$	$102 \pm 67$	na	na	$119 \pm 97$ $N\, \mathrm{mm}$	
PLL (C3/C4) ^c, e^	20	na	$19.8$	na	$8 \pm 4$	$54 \pm 25$	$111 \pm 49$	na	na	$151 \pm 84$ $N\, \mathrm{mm}$	
PLL (C2/C3) ^c, e^	20	na	$19.8$	na	$98 \pm 109$	$78 \pm 36$	$150 \pm 71$	na	na	$177 \pm 100$ $N\, \mathrm{mm}$	
ALL/PLL^c, e^ (mean)	20	na	$19.8$	na	$56 \pm 64$	$60 \pm 42$	$107 \pm 63$	na	na	$144 \pm 111$ $N\, \mathrm{mm}$	(Przybylski et al. 1996)
ALL ^a, c^	8	Yes	na	$3000$ DCM	$50 \pm 22$	$139 \pm 58$	$342 \pm 149$	$31.9 \pm 13.2$	$115 \pm 49$	Na	Mattucci et al. (2012)
			na	$120000$ DCM	$68 \pm 33$	$164 \pm 57$	$384 \pm 166$	$35.8 \pm 14.9$	$93 \pm 41$	Na	
			na	$\mathrm{900,000}$ DCM	$106 \pm 25$	$242 \pm 65$	$450 \pm 132$	$45.6 \pm 11.9$	$90 \pm 31$	Na	
PLL ^a, c^	8	Yes	na	$3000$ DCM	$63 \pm 22$	$215 \pm 68$	$341 \pm 104$	$29.3 \pm 12.1$	$76 \pm 31$	na	Mattucci et al. (2012)
			na	$\mathrm{120,000}$ DCM	$98 \pm 40$	$288 \pm 90$	$497 \pm 167$	$43.8 \pm 19.3$	$73 \pm 21$	na	
			na	$\mathrm{900,000}$ DCM	$142 \pm 69$	$362 \pm 151$	$437 \pm 135$	$39.4 \pm 15.2$	$65 \pm 20$	na	
CL ^a, c^	8	Yes	na	$3000$ DCM	$6.9 \pm 3.2$	$85 \pm 41$	$195 \pm 62$	$3.5 \pm 1.2$	$97 \pm 32$	na	Mattucci et al. (2012)
			na	$\mathrm{120,000}$ DCM	$10.1 \pm 3.4$	$122 \pm 43$	$270 \pm 91$	$6.0 \pm 2.2$	$112 \pm 51$	na	
			na	$\mathrm{900,000}$ DCM	$11.8 \pm 3.6$	$142 \pm 40$	$286 \pm 73$	$6.1 \pm 1.7$	$111 \pm 46$	na	
LF ^a^	10	Yes	na	$33$ SCM	na	na	na	$1.7 \pm 0.5$	$49.6 \pm 7.1$	na	Nachemson and Evans (1968)
					na	na	na	$8.9 \pm 0.9$	$62.5 \pm 7.5$	na	
					na	na	na	$2.2 \pm 0.1$	$33.0 \pm 1.0$	na	
					na	na	na	$1.1 \pm 0.9$	$21.6 \pm 64$	na	
					na	na	na	$3.3 \pm 0.4$	$80.0 \pm 12$	na	
					na	na	na	$2.3 \pm 0.3$	$68.5 \pm 1.5$	na	
					na	na	na	$1.6 \pm 0.1$	$32.8 \pm 0.3$	na	
					na	na	na	$2.5 \pm 1.1$	$43.3 \pm 4.3$	na	
LF ^a, c^	8	Yes	na	$3000$ DCM	$24.6 \pm 15.1$	$118 \pm 70$	$243 \pm 118$	$5.6 \pm 2.4$	$62 \pm 12$	na	Mattucci et al. (2012)
				$\mathrm{120,000}$ DCM	$28.6 \pm 13.3$	$141 \pm 65$	$328 \pm 121$	$8.0 \pm 3.1$	$58 \pm 13$	na	
				$\mathrm{900,000}$ DCM	$29.5 \pm 15.5$	$144 \pm 70$	$258 \pm 99$	$6.5 \pm 2.4$	$52 \pm 16$	na	
ISL^a, c^	8	Yes	na	$3000$ DCM	$13.7 \pm 10.0$	$12 \pm 8$	$56 \pm 37$	$4.5 \pm 2.9$	$65 \pm 17$	na	Mattucci et al. (2012)
				$\mathrm{120,000}$ DCM	$33.2 \pm 25.7$	$36 \pm 25$	$93 \pm 69$	$7.5 \pm 6.5$	$40 \pm 12$	na	
				$\mathrm{900,000}$ DCM	$29.9 \pm 15.0$	$35 \pm 17$	$98 \pm 66$	$8.3 \pm 6.2$	$45 \pm 12$	na	
IGHL^d, e^	7 (younger)	Yes	$50$	na	$125.3 \pm 24.3$	$77 \pm 5$	$874 \pm 70$	$7.4 \pm 0.8$	$16.6 \pm 1.5$	$5135 \pm 516$ $N\bullet \mathrm{mm}$	Lee et al. (1999)
	5 (older)	Yes			$67.8 \pm 9.9$	$61 \pm 7$	$535 \pm 28$	$15.8 \pm 4.1$	$10.1 \pm 1.0$	$3177 \pm 377$ $N\bullet \mathrm{mm}$	
AB-IGHL^d, e^	16	na	$2.4$	na	na	na	na	$5.5 \pm 2.0$	$34.0 \pm 10.5$	na	Bigliani et al. (1992)
AB-IGHL^d^	10	Yes	$10$	na	$14.8 \pm 13.1$	na	na	$0.8 \pm 0.4$	$33.3 \pm 23.6$	na	Moore et al. 2004)
PB-IGHL^d, e^	16	na	$2.4$	na	na	na	na	$5.6 \pm 1.9$	$23.1 \pm 4.6$	na	Bigliani et al. (1992)
PB-IGHL^d^	11	Yes	$10$	na	$31.5 \pm 12.7$	na	na	na	$22.8 \pm 11.1$	na	Moore et al. (2005)
SB-IGHL^d, e^	16	na	$2.4$	na	na	na	na	$5.6 \pm 1.9$	$24.0 \pm 6.2$	na	Bigliani et al. (1992)
AB-IGHL/PB-IGHL/SB-IGHL ^d, e^ (mean)	48	na	$2.4$	na	na	na	na	$5.5 \pm 2.2$	$27.0 \pm 8.9$	na	Bigliani et al. (1992)
CAL^b^ ^e^	10 (younger)	na	100	na	na	$51.6 \pm 24.7$	$351.8 \pm 47.2$	$32.7 \pm 7.6$	$19.9 \pm 5.9$	na	Fremerey et al. (2000)
	10 (older)	na			na	$38.6 \pm 18.9$	$279.6 \pm 39.4$	$31.8 \pm 8.5$	$22.2 \pm 8.6$	na	
Scapholunate Ligament ^c^ ^e^	16	Yes	$50$	na	na	$66.4 \pm 28.6$	$357 \pm 110$	na	$68.1 \pm 12.1$	na	Johnston et al. (2004)
USL	8	na	$5$	na	$13.2 \pm 1.4$	na	na	$6.3 \pm 0.9$	na	na	Martins et al. (2013)
RL	8	na	$5$	na	$6.8 \pm 1.3$	na	na	$3.4 \pm 0.8$	na	na	Martins et al. (2013)

^a^ Values referred to control group; ^b^ Values referred to the fresh samples group; ^c^ Values referred to the frozen samples group; ^d^ Values referred to the fresh frozen group; ^e^ Values referred to the bone–ligament–bone complex; ^f^ Values referred to a knee flexion of 90°; ^g^ Values referred to a knee flexion of 45°; ^h^ Values referred to a knee flexion of 30°; ^i^ Values referred to a knee flexion of 0°; ^j^ Values referred to a knee flexion of 15°; ^k^ Values referred to a knee flexion of 70°; ^l^ Values referred to a knee flexion of 60°; ^m^ Values referred to longitudinal specimens; ^n^ Values referred to transvers specimens; ^o^ Young’s modulus at 20% strain; ^p^ Young’s Modulus defined as the slope of the linear region of the stress–strain curve between 5 and 10% of strain; ^q^ Tangent modulus between 4 and 7% strain; ^r^ Elastic modulus was determined as the gradient of the line of best fit for the most linear portion of the stress/strain curve

### Comparison between the mechanical properties of animal and human ligaments

All the collected data reported in the previous tables were organised in different bar graphs. Each bar in the graphs represents the range of values assumed by a specific mechanical property analysed; the bar is delimited by the standard deviation (STD) values centred on the mean value of the data considered. In certain cases, the same reference provides several bars with different values because, in the same article, animals of different breeds, different sexes, different ages, or right/left limbs were studied. As a result, different values were obtained in the same article, although the type of sample preparation and strain/displacement rate were the same.

**Fig. 2:**
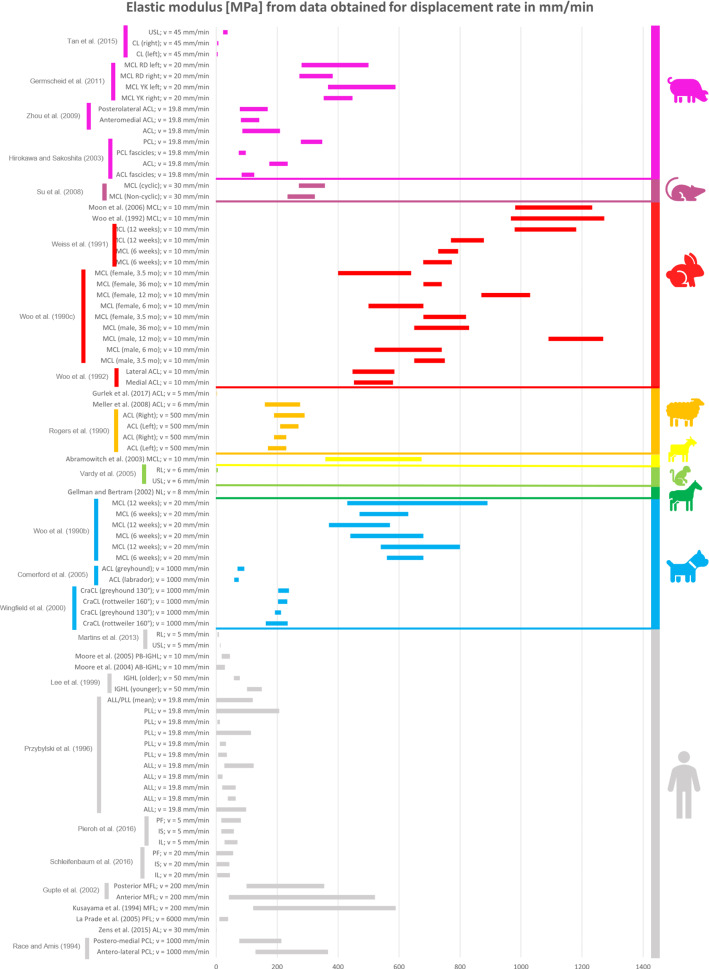
Young’s modulus for the considered animal species (mm/min)

**Fig. 3:**
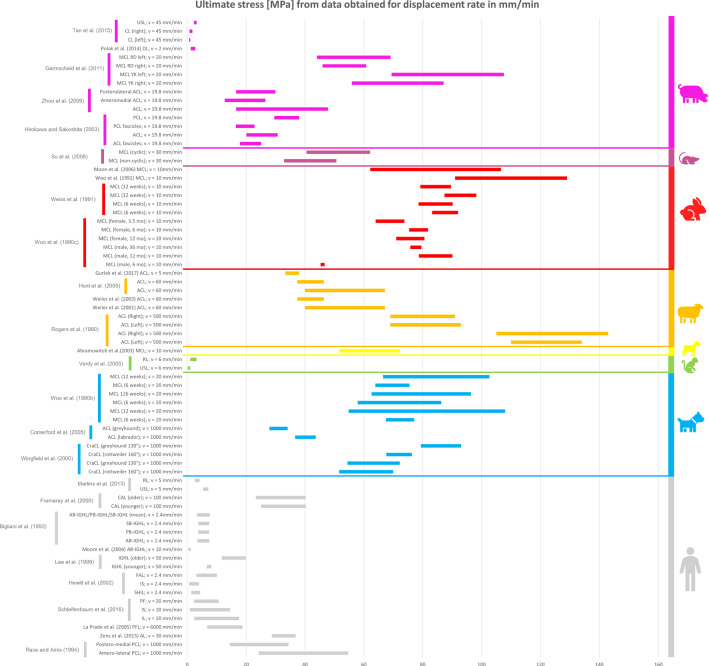
Ultimate tensile stress for the considered animal species (mm/min)

**Fig. 4:**
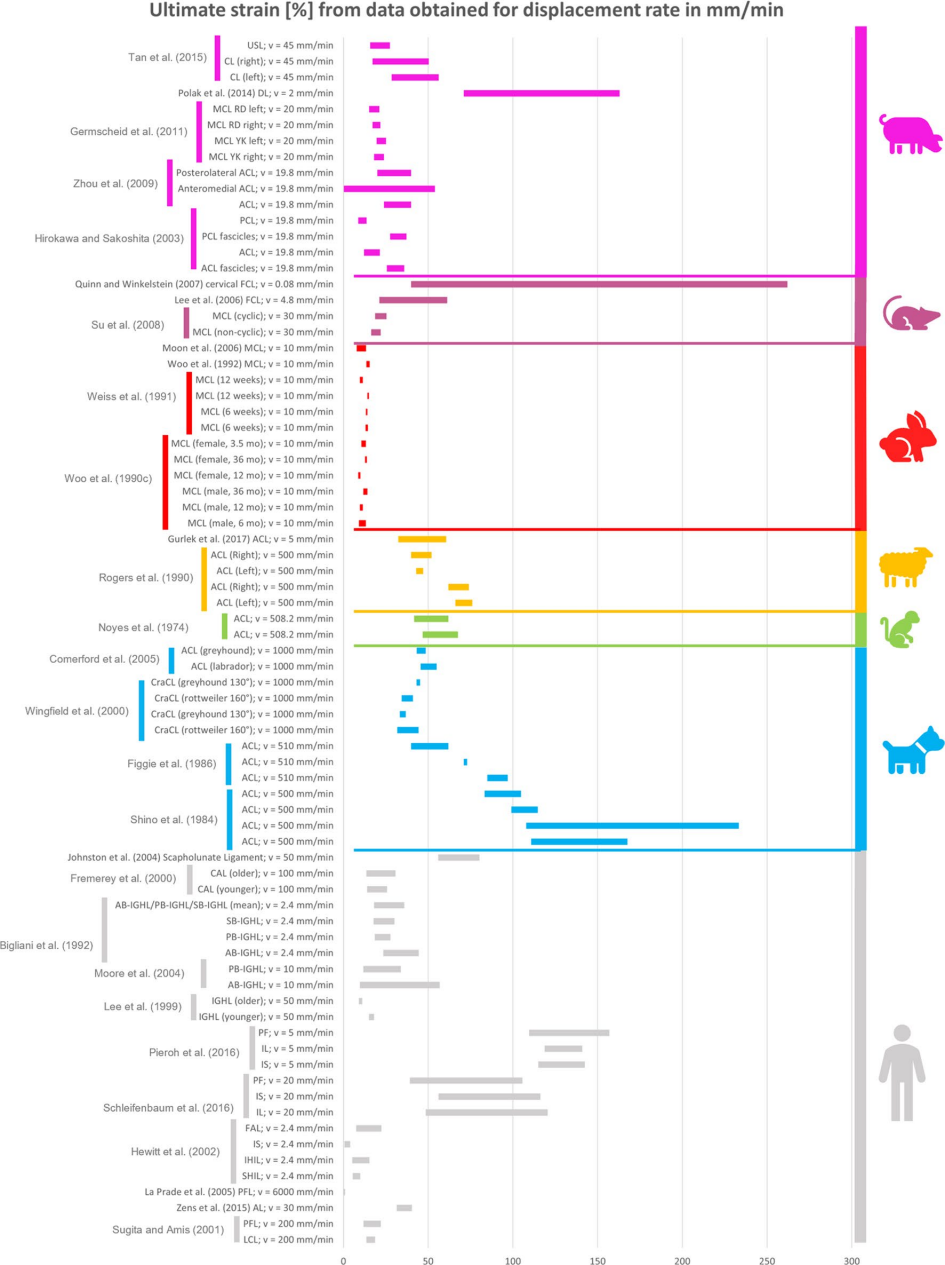
Ultimate strain for the considered animal species (mm/min)

**Fig. 5:**
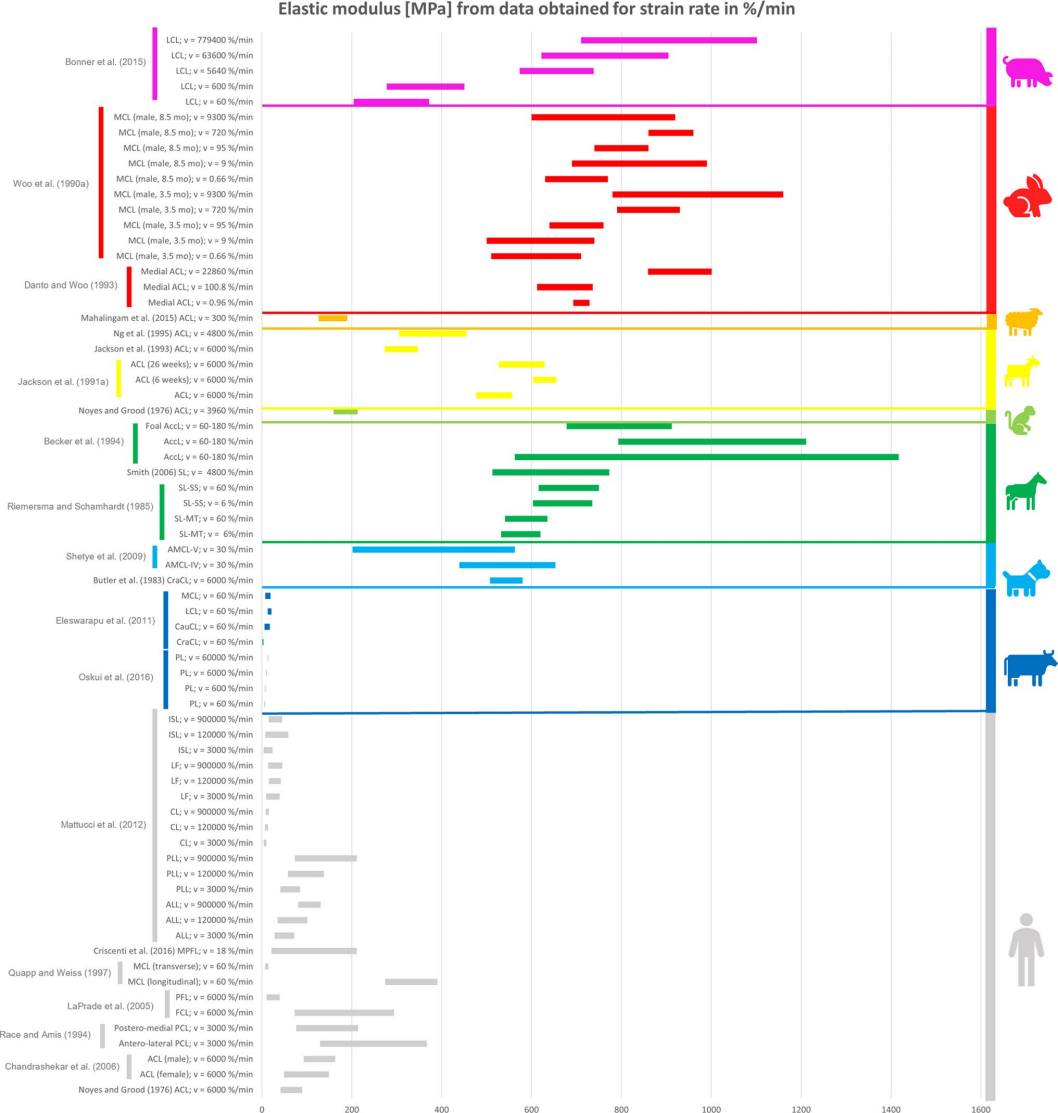
Young’s modulus for the considered animal species (%/min)

**Fig. 6:**
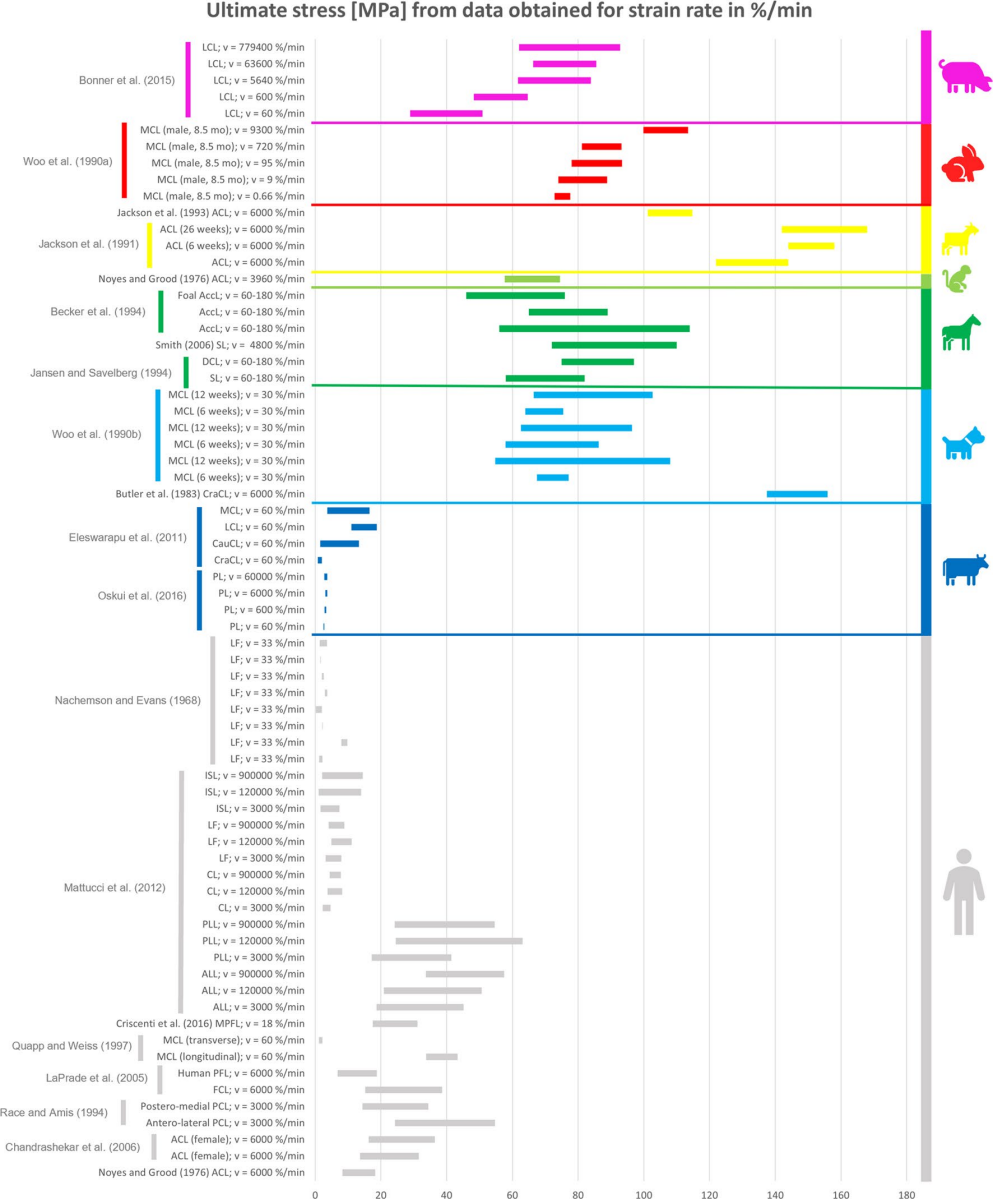
Ultimate tensile stress for the considered animal species (%/min)

**Fig. 7:**
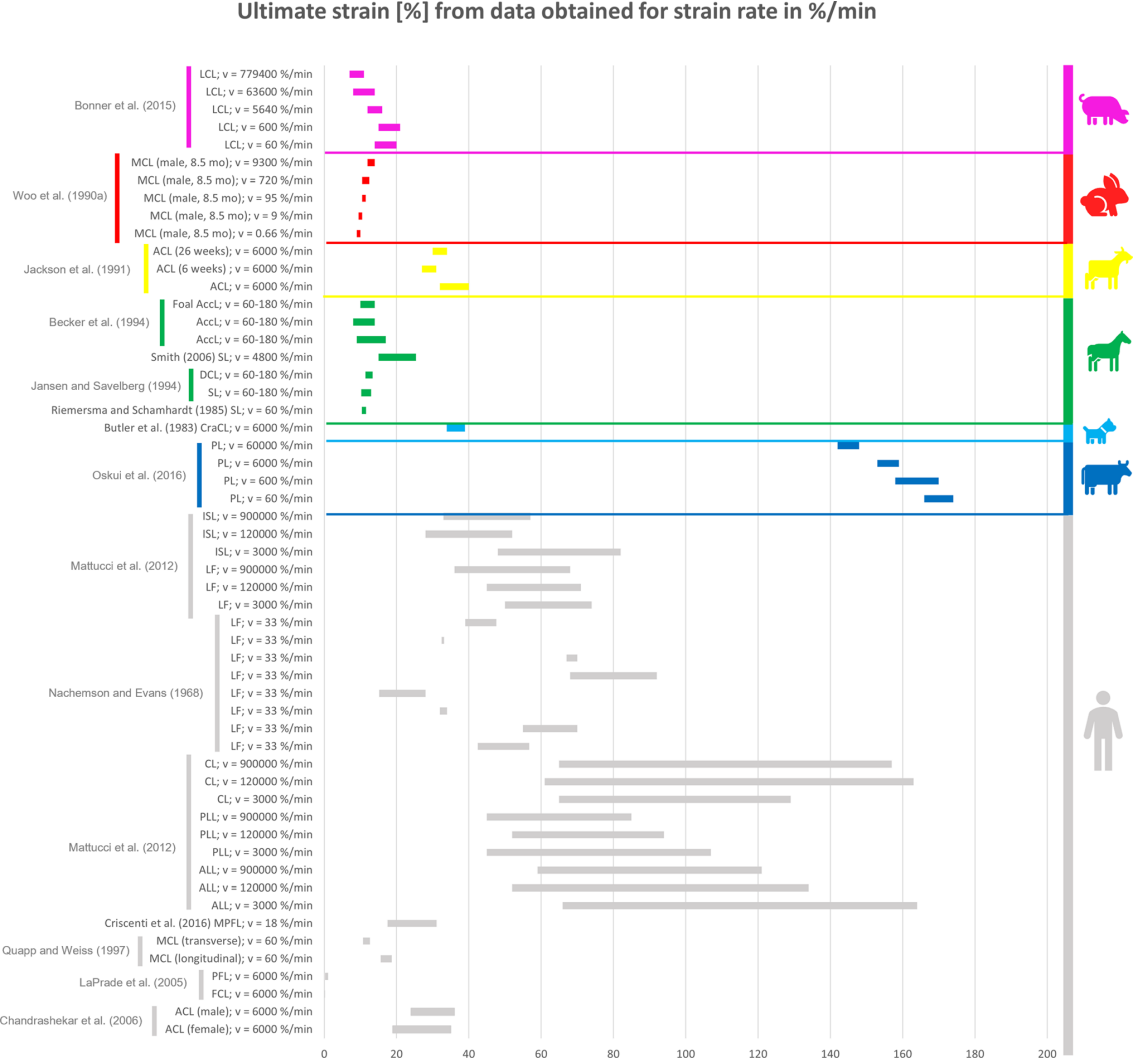
Ultimate strain for the considered animal species (%/min)

All the data reported in the previous tables were organised in different bar graphs. The elastic modulus, the ultimate tensile stress, and the ultimate strain report the strain rate in mm/min (Figs. 2, 3 and 4) and in %/min (Figs. 5, 6 and 7). For standardisation, values reported in mm/min and in cm/min have been modified to obtain values in mm/s. Data that did not report the strain rate values were not used for graphing and analysis. During the evaluation of all the articles related to rabbit ligaments, different MCL elastic modulus values were found. In particular, the article of Xie et al. (Xie et al. 2021) shows an MCl elastic modulus equal to 3 GPa, a greater value compared to the other articles. The high variability in the results may be due to the experimental setup, since they used a tension–torsion combined testing machine. Given that the elastic modulus value obtained by Xie et al. appears to be an outlier, this study was removed from our evaluation.

For better data visualisation and comparison of the mechanical properties of the ligaments between different animal species and the human, each species was associated with a specific colour: bovine (blue), dog (light blue), equine (green), monkey (light green), goat (yellow), sheep (orange), rabbit (red), rat (fuchsia). Regarding the mechanical properties of the human ligaments, grey was chosen.

### Results of mechanical property evaluation in mm/min

See Figs. 2, 3 and 4.

### Results of mechanical property evaluation in %/min

See Figs. 5, 6 and 7.

### Results of additional analysis-type of preconditioning

Table 5 reports the preconditioning that has been performed for different animal species and human.

**Table 5:** Type of preconditioning divided by animal species and human. ‘na’ indicates unavailable data

Animal species	Type of ligament	Type of preconditioning	Reference
Bovine	CraCL	30 loading cycles from 30 to 200 N at a quasi-static strain rate of 0.02 ${s}^{-1}$	Diotalevi et al. (2018)
	PL	20 tensile-compression cycles at the stretch of 1.3 and frequency of 1 Hz	Oskui et al. (2016)
Dog	ACL	4 loading/unloading cycles of 50 N and then a subfailure load (200 N), both at a slow deformation rate of 50 $\mathrm{mm}\bullet {\min}^{-1}$	Comerford et al. (2005)
	LCL, MCL, AMCL-IV, AMCL-V, PBL, PUL	10 cycles of 2% strain by use of a Haversine waveform	Shetye et al. (2009)
	MCL	10 cycles of approximately 2% strain at a rate of extension of 20 $\mathrm{mm}\bullet {\min}^{-1}$	Woo et al. (1990b)
Horse	SL, DCL	cyclically loaded 10 times to the level of the onset of the linear part of the force–displacement curve, which was drawn by a XY-recorder	Jansen and Savelberg (1994)
	AccL	10 times with a load of 50–125 *N*, depending on their size	Becker et al. (1994)
Foal	AccL	10 times with a load of 50–125 *N*, depending on their size	Becker et al. (1994)
Monkey	na	na	na
Mouse	na	na	na
Goat	ACL	10 cycles of 5% strain, at a speed of 2.5 s/cycle; after the last cycle, a resting load of 20 N was applied	Ng et al. (1995)
	MCL	10 cycles of loading between 0 and 2 mm of elongation for at 10 $\mathrm{mm}\bullet {\min}^{-1}$	Abramowitch et al. (2003)
Sheep	ACL	3 times of an AP force of ± 50 *N* was applied with a load displacement rate of 1 $\mathrm{mm}\bullet {s}^{-1}$	Weiler et al. (2004)
	ACL	10 cycles between 5 and 50 *N*	Viateau et al. (2013)
Rabbit	ACL	10 cycles between 0.0 and 0.3 mm extension (approximately 0 and 3% strain of the mid-substance of the ligament) at a rate of 10 $\mathrm{mm}\bullet {\min}^{-1}$	Woo et al. (1992)
	ACL	10 cycles from 0.0 to 0.3 mm elongation (approximately 0–3% strain of the mid-substance of the ligament), at a rate of 0.2 $\mathrm{mm}\bullet {s}^{-1}$	Danto and Woo (1993)
	ACL	21 cycles of stretching between 0 and 0.5 mm (approximately 5% strain) at 1 $\mathrm{mm}\bullet {s}^{-1}$ and on the 22nd cycle stretched until failure	Panjabi et al. (1996)
	PCL	10 cycles between 0 and 0.5 mm deformation at a rate of 10 $\mathrm{mm}\bullet {\min}^{-1}$	Murao et al. (1997)
	PCL	10 cycles between 0 and 0.5 mm deformation at a rate of 10 $\mathrm{mm}\bullet {\min}^{-1}$	Ma et al. (2009)
	LCL	3 cycles were performed by slowly cycling the ligament from its unloaded state just into the linear portion of its load-deformation response and then back to zero load	Tozilli and Arnoczky (1988)
	MCL	10 cycles of loading–unloading to 1 mm of elongation at a rate of 1 c $m\bullet {\min}^{-1}$	Woo et al. (1986)
	MCL	10 cycles of loading–unloading to 1 mm of elongation	Woo et al. (1990a)
	MCL	stretching the FMTC 10 times to the in situ strain level previously determined for each MCL specimen, at an elongation rate of 1 cm/min	Weiss et al. (1991)
	MCL	10 cycles between 0.0 and 0.5 mm extension (approximately 0 and 3% strain of the MCL substance, respectively) at an extension rate of 10 $\mathrm{mm}\bullet {\min}^{-1}$	Woo et al. (1992)
	MCL	10 cycles of between 0 and 1.5 mm of elongation	Moon et al. (2006)
	MCL	5 $min$ of a static preload of 0.5 N and then the maximum load was loaded and unloaded at a rate of 5 $\mathrm{mm}\bullet {\min}^{-1}$ at 0.5% of the maximum load 20 times	Xie et al. (2021)
Rat	MCL	5 cycles of load as low as the cyclic stretching and then stretched to failure immediately	Su et al. (2008)
	Thoracic FCL	30 cycles to 0.1 mm at 0.05 $mm\bullet {s}^{-1}$	Freedman et al. (2012)
	Cervical FCL	30 cycles to 0.2 mm (approximately 5% of load at gross failure)	Quinn and Winkelstein (2007)
Swine	LCL	5 cycles between 1 and 10 *N* at 10 $\mathrm{mm}\bullet {\min}^{-1}$, and repeated five times, then held at 0 N for 10 s	Bonner et al. (2015)
	MCL	2 cycles from − 20 *N* to + 8 N at 1 $\mathrm{mm}\bullet {\min}^{-1}$	Germscheid et al. (2011)
	MPFL	10 cycles of cyclic tension between 0 and 2 mm at an extension rate of 10 $\mathrm{mm}\bullet {\min}^{-1}$	Kim et al. (2014)
	CL	5 cycles from 0.25 to 1.0 *N* at 0.75 $\mathrm{mm}\bullet {s}^{-1}$	Tan et al. (2015)
Human	ACL, PCL, LCL, MCL	5 cycles to an intermediate load (approx. 147 *N*) at a strain rate of $5 \mathrm{cm}\bullet {\min}^{-1}$	Trent et al. (1976)
	ACL	20 cycles between 25 and 150 *N* tension at 0.25 Hz	Chandrashekar et al. (2006)
	LCL, PFL	Several cycles by slowly cycling the specimens from an unloaded state to the linear portion of their load deformation curve and back to zero load	LaPrade et al. (2005)
	LCL	5 loading cycles to a maximum load of 35 to 50 *N* tension at 0.5 Hz	Ciccone et al. (2006)
	LCL, MCL	10 cycles to a nominal 2 *N* and then to 3.5% strain at 1 Hz	Wilson et al. (2012)
	MCL	10 cycles to a maximum amplitude of 0.5 mm at a rate of 10 $\mathrm{mm}\bullet {\min}^{-1}$	Quapp and Weiss (1997)
	MCL, POL	10 cycles of 10 N to 50 *N* tension at 0.1 Hz	Wijdicks et al. (2010)
	MCL	10 cycles between 1 and 40 *N* tension at a crosshead speed of 10 $\mathrm{mm}\bullet {\min}^{-1}$	Robinson et al. (2005)
	MPFL	10 cycles to 3% of strain at a strain rate of 0.1%$\bullet {s}^{-1}$	Criscenti et al. (2016)
	MFL	10 cycles of 0–2 mm extension at a crosshead speed of 20 $\mathrm{mm}\bullet {\min}^{-1}$	Kusayama et al. (1994)
	MFL	10 load cycles resulting in 2 mm of extension at 20 $\mathrm{mm}\bullet {\min}^{-1}$	Gupte et al. (2002a)
	SHIL, IHIL, IS, FAL	10 cycles of loading to 5% strain	Hewitt et al. (2002)
	IL, IS, PF	crosshead displacement of 20 $\mathrm{mm}\bullet {\min}^{-1}$ and a maximum strain of 5%	Schleifenbaum et al. (2016)
	ALL, PLL, CL, LF, ISL	20 cycles of loading to 10% strain at a frequency of approximately 1 Hz	Mattucci et al. 2012)
	LF	5 load cycles were applied (from the unloaded condition) up to 9.8 *N* and subsequently to 19.6 *N*	Nachemson and Evans (1968)
	IGHL	10 cycles 1–2 mm at 50 $\mathrm{mm}\bullet {\min}^{-1}$	Lee et al. (1999)
	AB-IGHL	10 cycles between elongation limits of 0–0.3 mm at a rate of 10 $\mathrm{mm}\bullet {\min}^{-1}$	Moore et al. (2004)
	PB-IGHL	10 cycles between elongation limits of 0–0.3 mm at a rate of 10 $\mathrm{mm}\bullet {\min}^{-1}$	Moore et al. (2005)
	Scapholunate Ligament	25 times to 15% of their initial lengths at a rate of 66% of the initial lengths at a rate of 200 Hz	Johnston et al. (2004)

## Discussion

The mechanical properties evaluation of animal’s and human’s ligaments obtained from literature was performed in this review, considering the strain rate with two different units (mm/min and %/min). The analysis only dealt with the comparison between human and animal ligaments; thus, no comparison was performed among the mechanical properties of animal ligaments. From the analysis of the bar graphs, it was observed that generally, for each species, the values of the mechanical properties are included in a specific range. In particular, there is evidence that the value of strain rate has an effect on the mechanical properties of the ligaments (Pioletti et al. 1999). Differences in specimen behaviour at high and low strain rate values were shown in several papers. For instance, (Woo et al. 1990a) showed that the rabbit MCL ligament changes its properties at high strain rate values compared to low strain rate values (Figs. 5, 6 and 7). In other cases, for the same strain rate values, some mechanical properties show very different value as data obtained for rabbit MCL, v = 10 mm/min (Weiss et al. 1991) for elastic modulus (Fig. 2). Before the evaluation of the similarity between human ligaments and animal ligaments, it is important to specify that two different types of overlapping were found. The partial similarity means an overlapping between data, but the animal ligament shows a range of values that exceed human ligament values range. On the other hand, total similarity means that the animal ligaments show a range of values that is within the human ligament values range. The partial and total similarity between human and animal ligaments is reported in Appendix 1 and 2 in Supplementary material. Only the total similarity for all the parameters evaluated in this work is discussed in the following subsection, additionally, the percentage of overlap between the animal species and human ligament range was reported (%, of overlap between the distributions considered as the overlap with respect to the human values range).

### Evaluation of mechanical property

#### Evaluation of mechanical property in mm/min

Analysing the mechanical parameters obtained with a strain rate in mm/min (as reported in Figs. 2, 3 and 4), it can be observed that:

- Human AL (Zens et al. 2015) has a partial similarity for each animal ligament in terms of elastic modulus and ultimate stress. It has a verified total similarity of 38,7% in terms of ultimate strain with dog CraCL (Wingfield et al. 2000).
- Human AB-IGHL (Moore et al. 2005) has a total similarity with the swine CL (left) (Tan et al. 2015). This surrogate presents an error with respect to the human equal to 11.06% for elastic modulus, 52.50% for ultimate stress, and 58.89% for ultimate strain. Considering the elastic modulus, there are other total similarities: 17.09% with swine CL (right) (Tan et al. 2015) and 4.33% with sheep ACL (Gurlek et al. 2017). The ultimate stress presents only partial similarities. The ultimate strain presents total similarities with swine USL (Tan et al. 2015) of 24.57%, swine CL (right) of 70.33%, swine MCL (Germscheid et al. 2011) between 10.16% and 12.71%, swine posterolateral ACL (Zhou et al. 2009) of 42.37%, swine PCL of 20.76% and ACL between 19.91% and 21.61% (Hirokawa and Sakoshita 2003), and dog CraCL (Wingfield et al. 2000) between 7.33% and 26.52%.
- Human PB-IGHL (Moore et al. 2005) presents total similarities in terms of elastic modulus with swine USL of 58.09% (Tan et al. 2015). For the ultimate strain, there are total similarities with swine USL (Tan et al. 2015) of 52.25%, swine MCL (Germscheid et al. 2011) between 21.62% and 27.02%, swine ACL of 42.34% (Hirokawa and Sakoshita 2003), rat MCL (Su et al. 2008) between 25.22% and 30.63%, rabbit MCL (Weiss et al. 1991) between 4.50% and 6.30%, rabbit MCL (Woo et al. 1992) of 9.00%, and rabbit (female, 36 and 12 months) between 4.50% and 11.71% (Woo et al. 1990c).
- Human RL (Martins et al. 2013) has only partial similarities for all the parameters.
- Human ALL/PLL (mean) (Przybylski et al. 1996) presents total similarities in terms of elastic modulus with swine USL) (Tan et al. 2015) of 12.29%, dog ACL (Comerford et al. 2005) between 12.00% and 18,83%, swine ACL of 34.00%, and PCL of 19.33% (Hirokawa and Sakoshita 2003).
- Human USL (Martins et al. 2013) shows no similarities.
- Human IGHL (older) (Lee et al. 1999) presents a total similarity in terms of elastic modulus with dog ACL (Comerford et al. 2005) of 72.72%. For the ultimate stress, there are only partial similarities. For the ultimate strain, there is a total similarity with rabbit MCL (male,12 months) of 80% (Woo et al. 1990c).
- Human IGHL (younger) (Lee et al. 1999) presents only partial similarities for elastic modulus and ultimate strain.
- Human PF (Pieroh et al. 2016) presents a total similarity in terms of elastic modulus with dog ACL (Comerford et al. 2005) of 22.42%. For the ultimate strain, there are only partial similarities.
- Human IS (Pieroh et al. 2016) presents a total similarity in terms of elastic modulus with swine USL (Tan et al. 2015) of 36.16%. For the ultimate strain, there are only partial similarities.
- Human IL (Pieroh et al. 2016) presents a total similarity in terms of elastic modulus with swine USL (Tan et al. 2015) of 34.47%. For the ultimate strain, there are only partial similarities.
- Human PF (Schleifenbaum et al. 2016) presents a total similarity for elastic modulus of swine USL (Tan et al. 2015) of 26.49%. For the ultimate strain, there are total similarities with swine CL(left) (Tan et al. 2015) of 53.29%, swine posterolateral ACL (Zhou et al. 2009) of 37.86%, sheep ACL (right and left) (Rogers et al. 1990) between 44.49% and 71.94%, monkey ACL (Noyes and Grood 1976b) between 58.68% and 63.89%, and dog ACL (Comerford et al. 2005) between 46.00% and 52.06%.
- Human IS (Schleifenbaum et al. 2016) presents a total similarity in terms of elastic modulus with swine USL (Tan et al. 2015) of 34.96%. For the ultimate strain, there are total similarities with sheep ACL (right and left) (Rogers et al. 1990) between 16.66% and 20.00%, dog ACL (Figgie et al. 1986) between 3.33% and 20.00%, and dog ACL (Shino et al. 1984) between 26.00% and 36.00%.
- Human IL (Schleifenbaum et al. 2016) presents a total similarity in terms of elastic modulus with swine USL (Tan et al. 2015) of 35.13%. For the ultimate strain, there are total similarities with sheep ACL (right and left) (Rogers et al. 1990) between 13.88% and 16.66%, dog ACL (Figgie et al. 1986) between 2.77% and 16.66%, and dog ACL (Shino et al. 1984) between 21.66% and 30.00%.
- Human posterior MFL (Gupte et al. 2002) presents total similarities in terms of elastic modulus with dog CraCL (Wingfield et al. 2000) between 7.89% and 28.19%, sheep ACL (Meller et al. 2008) of 45.41%, sheep ACL (right and left) (Rogers et al. 1990) between 15.68% and 39.21%, rat MCL (Su et al. 2008) of 35.29%, and swine ACL of 23.45% and PCL of 27.45% (Hirokawa and Sakoshita 2003).
- Human anterior MFL (Gupte et al. 2002) presents total similarities in terms of elastic modulus with sheep ACL (Meller et al. 2008) of 24.16%, sheep ACL (right and left) (Rogers et al. 1990) between 8.34% and 20.86%, rat MCL (Su et al. 2008) between 17.94% and 18.78%, swine MCL (Germscheid et al. 2011) between 19.82% and 45.86%, swine ACL (Zhou et al. 2009) of 25.77%, swine posterolateral ACL (Zhou et al. 2009) of 19.01%, swine anteromedial ACL (Zhou et al. 2009) of 12.55%, swine PCL between 4.86% and 14,60%, and ACL between 8.51% and 12.47% (Hirokawa and Sakoshita 2003).
- Human MFL (Kusayama et al. 1994a) presents total similarities in terms of elastic modulus with dog CraCL (Wingfield et al. 2000) between 25.64% and 55.55%, sheep ACL (Meller et al. 2008) of 21.36%, sheep ACL (right and left) (Rogers et al. 1990) between 4.29% and 15.36%, rabbit MCL (Woo et al. 1992) medial of 51.28% and lateral of 67.52%, rat MCL (Su et al. 2008) between 38.46% and 47.00%, swine MCL (Germscheid et al. 2011) between 18.37% and 29.48%, and swine ACL of 23.24% and PCL of 46.96% (Hirokawa and Sakoshita 2003).
- Human PFL (LaPrade et al. 2005) presents a total similarity in terms of elastic modulus with swine USL (Tan et al. 2015) of 50.88%.
- Human antero-lateral PCL (Race and Amis 1994) presents total similarities in terms of elastic modulus with dog CraCL (Wingfield et al. 2000) between 8.45% and 30.21%, rat MCL (Su et al. 2008) between 36.16% and 37.81%, sheep ACL (right and left) (Rogers et al. 1990) between 16.80% and 42.01%, and swine ACL of 25.12% and PCL of 29.41% (Hirokawa and Sakoshita 2003). For the ultimate stress, there are total similarities with dog ACL (Comerford et al. 2005) between 20.39% and 23,02%, sheep ACL (Hunt et al. 2005) of 29.60%, sheep ACL (Weiler et al. 2004) of 29.60%, rat MCL (Su et al. 2008) of 58.55%, and swine PCL (Hirokawa and Sakoshita 2003) of 27.63%.
- Human postero-medial PCL (Race and Amis 1994) presents total similarities in terms of elastic modulus with dog CraCL (Wingfield et al. 2000) of 14.57%, swine ACL (Zhou et al. 2009) of 89.49%, swine posterolateral ACL (Zhou et al. 2009) of 66.01%, swine anteromedial ACL (Zhou et al. 2009) of 43.60%, and swine ACL (Hirokawa and Sakoshita 2003) of 29.56%. For the ultimate stress, there are total similarities with swine ACL between 36.00% and 53.00%, PCL of 32.00% (Hirokawa and Sakoshita 2003), and swine posterolateral ACL (Zhou et al. 2009) of 66.60%.
- Human Cal (older) (Fremerey et al. 2000) presents total similarities in terms of ultimate stress with dog ACL (Comerford et al. 2005) of 36.47%, sheep ACL (Gurlek et al. 2017) of 27.53%, and swine PCL (Hirokawa and Sakoshita 2003) of 49.41%. For the ultimate strain, there are total similarities with swine USL (Tan et al. 2015) of 67.44%, and rat MCL (Su et al. 2008) between 32.55% and 39.53%.
- Human Cal (younger) (Fremerey et al. 2000) presents total similarities in terms of ultimate stress with dog ACL (Comerford et al. 2005) of 40.78%, sheep ACL (Gurlek et al. 2017) of 30.78%, and swine PCL (Hirokawa and Sakoshita 2003) of 55.26%. For the ultimate strain, there are total similarities with swine MCL (Germscheid et al. 2011) between 40.67% and 50.84%.
- Human AB-IGHL/PB-IGHL/SB-IGHL (mean) (Bigliani et al. 1992) presents total similarities in terms of ultimate strain with swine MCL (Germscheid et al. 2011) between 31.46% and 33.70%, and rat MCL (Su et al. 2008) of 38.20%.
- Human FAL (Hewitt et al. 2002) presents total similarities in terms of ultimate strain with swine MCL (Germscheid et al. 2011) between 32.00% and 40.00%, swine ACL of 62.66% and PCL of 33.33% (Hirokawa and Sakoshita 2003), rat MCL (Su et al. 2008) of 37.33%, rabbit MCL (Moon et al. 2006) of 37.33%, rabbit MCL (Weiss et al. 1991) between 6.66% and 10.66%, rabbit MCL (Woo et al. 1992) of 13.33%, rabbit female (from 6 to 36 months) between 6.66% and 17.33% and rabbit male (from 6 to 36 months) between 10.66% and 28.00% (Woo et al. 1990c).
- Human IHIL (Hewitt et al. 2002) presents total similarities in terms of ultimate strain with swine PCL of 50.00% (Hirokawa and Sakoshita 2003), rabbit MCL (Moon et al. 2006) of 56.00%, rabbit MCL (Weiss et al. 1991) between 10.00% and 14.00%, and rabbit female (from 6 to 36 months) between 10.26% and 26.00% and rabbit male (from 6 to 36 months) between 16.00% and 42.00% (Woo et al. 1990c).
- Human IS (Hewitt et al. 2002) presents total similarities in terms of ultimate stress with monkey RL (Vardy et al. 2005) of 65.08%, and swine CL of 28.99% (right) and 12.42% (left) (Tan et al. 2015).
- Human SHIL (Hewitt et al. 2002) presents total similarity in terms of ultimate stress with swine USL of 28.94% (Tan et al. 2015), and swine DL (Polak et al. 2014) of 50.00%.
- Human Scapholunate Ligament presents total similarities in terms of ultimate strain with sheep ACL (right and left) (Rogers et al. 1990) between 41.32% and 49.58%, and dog ACL (Figgie et al. 1986) of 8.26%.
- Human PFL (Sugita and Amis 2001) presents total similarities in terms of ultimate strain with swine MCL (Germscheid et al. 2011) between 46.15% and 57.69%, swine PCL of 90.38% (Hirokawa and Sakoshita 2003), rat MCL (Su et al. 2008) of 53.84%, rabbit MCL (Woo et al. 1992) of 19.23%, rabbit MCL (Weiss et al. 1991) between 9.61% and 13.46%, rabbit female (12 months) of 9.61% and rabbit male (36 months) of 23.07% (Woo et al. 1990c).
- Human LCL (Sugita and Amis 2001) presents total similarities in terms of ultimate strain with rabbit MCL (Woo et al. 1992) of 40.00%, and rabbit MCL (Weiss et al. 1991) of 20%.

#### Evaluation of mechanical property in %/min

Analysing the mechanical parameters obtained with a strain rate in %/min in Figs. 5, 6 and 7, it can be observed that:

- Human ACL (Noyes and Grood 1976) has no similarities for elastic modulus. For the ultimate stress, there are only partial similarities with calf CauCL, LCL and MCL (Eleswarapu et al. 2011).
- Human ACL (Chandrashekar et al. 2006) has only partial similarities for elastic modulus and ultimate stress. Instead, for the ultimate strain, there are total similarities with goat ACL (Jackson et al. 1991) between 25% and 33.33%.
- Human anterolater PCL (Race and Amis 1994) presents total similarities in terms of elastic modulus with monkey ACL (Noyes and Grood 1976) of 21.82% and goat ACL (Jackson et al. 1993) of 31.09%. For the ultimate stress, there is total similarities with swine LCL (Bonner et al. 2015) of 72.36%. For the ultimate strain, there is a total similarity with swine LCL (Bonner et al. 2015) of 56.60%.
- Human posteromedial PCL (Race and Amis 1994) presents total similarities in terms of elastic modulus with monkey ACL (Noyes and Grood 1976) of 37.68% and sheep ACL (Mahalingam et al. 2015) of 46.37%. For the ultimate strain, there are total similarities with swine LCL (Bonner et al. 2015) of 55.55%, and with equine SL (Smith 2006) of 96.29%.
- Human FCL (LaPrade et al. 2005) presents total similarities in terms of elastic modulus with monkey ACL (Noyes and Grood 1976) of 23.48% and sheep ACL (Mahalingam et al. 2015) of 28.90%.
- Human MCL (longitudinal) (Quapp and Weiss 1997) presents only partial similarities for elastic modulus and ultimate strain.
- Human MCL (transverse) (Quapp and Weiss 1997) presents total similarities in terms of elastic modulus with cow PL (Oskui et al. 2016) at different strain rate values, 0.28% (600%/min), 7.56% (6000%/min), and 9.80% (60,000%/min).
- Human MPFL (Criscenti et al. 2016) presents total similarity in terms of ultimate stress with calf MCL (Eleswarapu et al. 2011). For ultimate strain, there is a total similarity with goat ACL (Jackson et al. 1991) of 29.48%.
- Human ALL (strain rate of 3000%/min) (Mattucci et al. 2012) presents a total similarity in terms of ultimate strain with cow PL (Oskui et al. 2016) at strain rate of 6000%/min and 60,000%/min of 6.12%.
- Human ALL (strain rate of 12,000%/min) (Mattucci et al. 2012) presents only partial similarities for ultimate stress.
- Human ALL (strain rate of 900,000%/min) (Mattucci et al. 2012) presents only partial similarities for ultimate stress.
- Human PLL (strain rate of 3000%/min) (Mattucci et al. 2012) presents only partial similarities for ultimate stress.
- Human PLL (strain rate of 12,000%/min) (Mattucci et al. 2012) presents total similarities in terms of ultimate stress with PL (Oskui et al. 2016) of 0.44% (60%/min), 0.82% (600%/min), 0.98% (6000%/min), 1.45% (60,000%/min), with calf CraCL of 1.9%, CauCL of 18.70%, LCL of 12.36%, MCL of 20.28% (Eleswarapu et al. 2011), and swine LCL (Bonner et al. 2015) of 34.86%. For the ultimate strain, there are a total similarities with dog CraCL (Butler et al. 1983) of 5.31%, equine SL (Riemersma and Schamhardt 1985) of 1.27%, equine SL of 2.97% and DCL of 2.12% (Jansen and Savelberg 1994), equine SL (Smith 2006) of 11.06%, equine AccL (Becker et al. 1994) between 4.25% and 6.38%, goat ACL (Jackson et al. 1991) between 4.25% and 8.50%, rabbit MCL (Woo et al. 1990a) between 1.06% and 2.12%, rabbit MCL (Moon et al. 2006) of 5.95%, and swine LCL (Bonner et al. 2015) between 4.25% and 6.38%.
- Human PLL (strain rate of 900,000%/min) (Mattucci et al. 2012) presents a total similarity in terms of elastic modulus with sheep ACL (Mahalingam et al. 2015) of 46.37%. For ultimate stress, there is a total similarity with swine LCL (Bonner et al. 2015) of 72.36%.
- Human CL (strain rate of 3000%/min) (Mattucci et al. 2012) presents total similarities in terms of elastic modulus with cow PL (Oskui et al. 2016) at different strain rate values, of 1.25% (60%/min), 0.31% (600%/min). For ultimate stress, there are similarities with cow PL (Oskui et al. 2016) at different strain rate values, of 11.66% (60%/min), 21.66% (600%/min), 25.83% (6000%/min), and 38.33% (60,000%/min).
- Human CL (strain rate of 12,000%/min) (Mattucci et al. 2012) presents total similarities in terms of elastic modulus with cow PL (Oskui et al. 2016) at different strain rate, of 0.29% (600%/min), 7.94% (6000%/min). There are only partial similarities for ultimate strain with cow PL (Oskui et al. 2016) with strain rate of 600, 6000 and 60,000%/min.
- Human CL (strain rate of 900,000%/min) (Mattucci et al. 2012) presents total similarities in terms of elastic modulus with cow PL (Oskui et al. 2016) at different strain rate values, of 0.29% (600%/min), 7.94% (6000%/min), and 7.94% (60,000%/min).
- Human LF (strain rate of 3000%/min) (Mattucci et al. 2012) presents total similarities in terms of elastic modulus with cow PL (Oskui et al. 2016) at different strain rate, of 1.79% (6000%/min), 2.31% (60,000%/min), and calf LCL (Eleswarapu et al. 2011) of 26.95%.
- Human LF (strain rate of 12,000%/min) (Mattucci et al. 2012) presents only partial similarities for elastic modulus and ultimate stress.
- Human LF (strain rate of 900,000%/min) (Mattucci et al. 2012) presents only partial similarities for elastic modulus and ultimate stress.
- Human ISL (strain rate of 3000%/min) (Mattucci et al. 2012) presents total similarities in terms of elastic modulus with calf LCL of 40.7%, CauCL of 59% (Eleswarapu et al. 2011), and with cow PL (Oskui et al. 2016) at different strain rate, of 0.40% (60%/min), 0.1% (600%/min), 2.70% (6000%/min) and 3.50% (60,000%/min). For ultimate stress, there are total similarities with cow PL (Oskui et al. 2016) at different strain rate values, of 4.82% (60%/min), 8.96% (600%/min), 10.69% (6000%/min), and 15.86% (60,000%/min).
- Human ISL (strain rate of 12,000%/min) (Mattucci et al. 2012) presents total similarities in terms of elastic modulus with calf CauCL of 90.77% (Eleswarapu et al. 2011), and with cow PL (Oskui et al. 2016) at different strain rate, of 2.15% (60%/min), 4.00% (600%/min), 4.76% (6000%/min) and 7.36% (60,000%/min). For ultimate stress, there are total similarities with calf LCL of 90.76%, CauCL of 59% (Eleswarapu et al. 2011), and with cow PL (Oskui et al. 2016) at different strain rate, of 2.15% (60%/min), 4.00% (600%/min), 4.76% (6000%/min), and 7.07% (60,000%/min). For the ultimate strain, there are total similarities with dog CraCL (Butler et al. 1983) of 20.83%, goat ACL (Jackson et al. 1991) between 16.66% and 33.33%.
- Human ISL (strain rate of 900,000%/min) (Mattucci et al. 2012) presents total similarities in terms of ultimate stress with calf CauCL of 95.16% (Eleswarapu et al. 2011), and cow PL (Oskui et al. 2016) at different strain rate, of 2.25% (60%/min), 4.19% (600%/min), 5.00% (6000%/min), and 7.41% (60,000%/min). For the ultimate strain, there is a total similarity with dog CraCL (Butler et al. 1983) of 20.83%.
- Human LF(Nachemson and Evans 1968), presents total similarities in terms of the ultimate stress with calf CraCL (Eleswarapu et al. 2011) from 32.42 to 66.66%, and cow PL (Oskui et al. 2016) at different strain rate, of 12.72% (60%/min), 23.63% (600%/min), and 41.81% (60,000%/min). For ultimate strain, specimens with an average ultimate stress of 21.60 MPa have a total similarity with equine SL (Smith 2006) of 81.25%.

### Type of preconditioning

The preconditioning consists typically of 10/20 cycles of loading/unloading until a certain value or inside an interval of tension or deformation. As can be seen in Table 5, in the majority of the reviewed articles, the specimens underwent preconditioning by 10 cycles of approximately 0–5% strain (Shetye et al. 2009, Woo et al. 1990b, Ng et al. 1995, Woo et al. 1992, Danto and Woo 1993, Murao et al. 1997, Ma et al. 2009, Moon et al. 2006, Kim et al. 2014, Wilson et al. 2012, Quapp and Weiss 1997, Criscenti et al. 2016, Kusayama et al. 1994, Hewitt et al. 2002, Schleifenbaum et al. 2016, Moore et al. 2004 and Moore et al. 2005) or around 50 N (Becker et al. 1994, Viateau et al. 2013, Wijdicks et al. 2010 and Robinson et al. 2005). It is also possible to observe that in many cases (Diotalevi et al. 2018, Woo et al. 1990b, Abramowitch et al. 2003, Weiler et al. 2004, Woo et al. 1992, Panjabi et al. 1996, Woo et al. 1986, Weiss et al. 1991, Xie et al. 2021, Kim et al. 2014, Tan et al. 2015 and Schleifenbaum et al. 2016) the loading/unloading cycles are performed at the same strain rate used during the tensile tests. Lastly, it can be said that the type of preconditioning varies with different ligaments in various animal species and human specimens. In fact, it is important to point out that in general there is no standardisation in terms of the number of cycles and the value of deformation or tension at which the preconditioning is performed.

### Limitations

The individuation from the existing scientific literature of the most suitable surrogate to imitate the behaviour of human ligaments is hampered by several inhomogeneities in the experimental test protocol. This study also did not consider parameters such as animal age, sex, and lifetime activity. These parameters may influence the biomechanical characteristics of soft tissues. Additionally, the comparison of ligaments should be conducted by evaluating their composition. Future studies should compare the influence of these parameters on the mechanical properties of animal and human tendons, which would lead to a more accurate assessment of the ligament to be used for ex vivo testing. Moreover, here the mechanical properties of knee animals and human ligaments were reported evaluating only a uniaxial tensile test condition. Further studies will be needed to analyse their mechanical behaviour at different angles.

## Conclusions

This systematic review aimed at defining the most suitable surrogates for mimicking the behaviour of human ligaments when subjected to uniaxial tensile tests. For this reason, the scientific literature was reviewed, evaluating the experimental studies involving the mechanical properties of animal ligaments. Differences and similarities between human and animal ligaments were highlighted and commented upon and the best candidates were determined and discussed. The comparison between the mechanical properties of animal ligaments highlighted how they cannot always be compared with their human counterparts; on the other hand, there are many similarities between different anatomical parts. In general, no specific animal ligaments can provide a suitable model for its respective human counterpart concerning all the three primary mechanical properties (Young modulus, ultimate tensile stress, and ultimate tensile strain) at the same strain rate. It is interesting to note that in the current scientific literature, different animal models (bovine, dog, rabbit, and swine) were adopted to evaluate the knee repair technologies; nevertheless, despite this wide use, no clear similarities were found in their mechanical properties. Further studies will be needed to further compare the mechanical properties of these ligaments and ensure that the scientific evidence derived from such experimental studies can be considered reliable.

Several similarities were observed in some properties between animal and human ligaments. These similarities were found despite the ligaments having been analysed at different strain rates. The results showed similarities between animal and human ligaments that should be considered in the evaluation of scaffolds and sutures.

Considering the results reported for tests performed in mm/min:

- Swine CL with a displacement rate of 45 mm/min is comparable (total similarity in terms of elastic modulus, ultimate tensile stress and ultimate strain) with human AB-IGHL with a displacement rate of 10 mm/min;
- Swine USL with a displacement rate of 45 mm/min is comparable (total similarity in terms of elastic modulus and ultimate strain but not for ultimate stress) with human PB-IGHL with a displacement rate of 10 mm/min;
- Swine ACL and posterolateral ACL with a displacement rate of 19.8 mm/min are comparable (total similarity in terms of elastic modulus and ultimate strain but not for ultimate stress) with human posteromedial PCL with a displacement rate of 1000 mm/min;
- Rat MCL with a displacement rate of 30 mm/min is comparable (total similarity in terms of elastic modulus and ultimate stress but not for ultimate strain) with human posteromedial PCL with a displacement rate of 1000 mm/min;
- Swine PCL with a displacement rate of 19.8 mm/min is comparable (total similarity in terms of elastic modulus and ultimate stress but not for ultimate strain) with human anterolateral PCL with a displacement rate of 1000 mm/min;

It’s important mentioning that monkey RL with a displacement rate of 6 mm/min has a partial similarity with human RL with a displacement rate of 5 mm/min for elastic modulus and ultimate tensile stress. This result should be further analysed in future works.

Considering the results reported for tests performed in %/min:

- Swine LCL with a strain rate of 60%/min is comparable (total similarity in terms of ultimate stress and ultimate strain but not for elastic modulus) with human anterolateral PLL with a strain rate of 12,000%/min;
- Swine LCL with a strain rate of 6o %/min and 600%/min are comparable (total similarity in terms of ultimate stress and ultimate strain but not for elastic modulus) with human anterolateral PCL with a strain rate of 3000%/min;
- Swine LCL with a strain rate of 6o %/min is comparable (total similarity in terms of ultimate stress and ultimate strain but not for elastic modulus) with human PLL with a strain rate of 12,000%/min;
- Cow PL with a strain rate of 6o %/min and 600% is comparable (total similarity in terms of elastic modulus and ultimate stress but not for ultimate strain) with human CL with a strain rate of 3000%/min. Moreover, the cow PL at different strain rate shows some partial similarities with human CL with a strain rate of 900,000%/min;
- Cow PL with a different strain rate is comparable (total similarity in terms of elastic modulus and ultimate stress but not for ultimate strain) with human ISL with strain rates of 3000%/min and 12,000%/min. The human ISL (3000%/min and 12,000%/min) shows some partial similarities with calf CauCL for elastic modulus and ultimate stress. Moreover, increasing the strain rate, some partial similarities with cow PL remain.

In our previous review, similarities between human, swine, equine, rabbit, rat, and goat tendons were found and discussed in detail. Here, the analysis of the mechanical properties for human and animal ligaments reported similarities between human and swine, cow, and rat ones. Comparing these two reviews, it can be stated that there are similarities between the mechanical properties of human and animals’ tendons and ligaments. In particular, the species with most similarities for both tendons and ligaments are swine and rat. These results may pave the way for future works.

As a concluding remark, it seems highly probable that the choice of parameter setting significantly affects the results of the experimental studies reviewed and discussed here. Unfortunately, different authors reported their results with different settings. The lack of standard test settings (strain rate, pre-conditioning) for the experiments should be considered when interpreting the results reported in the scientific literature. Future studies will be needed to evaluate ligaments from different animals and anatomical regions with the same test conditions and strain rate, in a fully comparable way. Based on the evaluation of mechanical characterisation of ligaments analysed in this work, the authors thought the following suggestions for best practices. After the tendon extraction from the anatomical site, it is important to use the same protocol for each of them. It is advisable to not perform the test on frozen samples. However, in case of frozen samples, the defrosting process should be done at least 24 h before the tests. Furthermore, before the test, the specimens’ thickness and width should be measured. These measurements can be done either in a normal condition or with a preload. The preload value should be evaluated based on the literature information; if no data are available, the preload should not exceed 10 Newton. Of course, all the parameters used for the test should be fully reported in the article and defined after an evaluation of the literature on the specific tissue. Based on this review, the standard preconditioning for ligaments should be 20 cycles at 1%/s of strain rate (starting from the preload force). Finally, the range where Young’s modulus was calculated should be reported in the article.

### Supplementary Information

Below is the link to the electronic supplementary material.

Supplementary file1 (DOCX 23 kb)

Supplementary file2 (DOCX 33 kb)
