# Supplementary material for: Mechanical properties of animal ligaments: a review and comparative study for the identification of the most suitable human ligament surrogates
Source: Biomech Model Mechanobiol. 2023 May 11;22(5):1645–83. doi: 10.1007/s10237-023-01718-1 (PMC10511400; doi:10.1007/s10237-023-01718-1)
Supplement: Supplementary file 1 — Supplementary file1 (DOCX 23 kb) [file 10237_2023_1718_MOESM1_ESM.docx]

| **Reference** | **Elastic Modulus** | **Ultimate Stress** | **Ultimate strain** |
| --- | --- | --- | --- |
| **AL; v = 30 mm/min (Zens et al. (2015))** | **Monkey RL; v = 6 mm/min (Vardy et al. (2005))** | **Sheep ACL; v = 5 mm/min (Gurlek et al. (2017))**  **Rat MCL; v = 30 mm/min (Su et al. (2008))**  **Swine Posterolateral ACL and ACL; v = 19,8 mm/min (Zhou et al. (2009))**  **Swine PCL; v = 19.8 mm/min (Hirokawa and Sakoshita (2003))** | **Swine CL (Right and left); v = 45 mm/min (Tan et al. (2015))**  **Swine Anteromedial ACL, Posterolateral ACL and ACL; v = 19,8 mm/min (Zhou et al. (2009))**  **Swine ACL; v = 19.8 mm/min (Hirokawa and Sakoshita (2003))**  **Rat FCL; v = 4,8 mm/min (Lee et al. (2006))**  **Sheep ACL; v = 5 mm/min (Gurlek et al. (2017))**  **Dog CraCL; v = 1000 mm/min (Wingfield et al. (2000))** |
| **AB-IGHL; v = 10 mm/min (Moore et al. (2004))** | **Sheep ACL; v = 5 mm/min (Gurlek et al. (2017))**  **Monkey RL; v = 6 mm/min (Vardy et al. (2005))**  **Swine CL (right and left) and USL v = 45 mm/min (Tan et al. (2015))** | **Monkey USL; v = 6 mm/min (Vardy et al. (2005))**  **Swine CL (right and left); v = 45 mm/min (Tan et al. (2015))** | **Swine USL and CL (right and left); v = 45 mm/min (Tan et al. (2015))**  **Swine MCL; v = 20 mm/min (Germscheid et al. (2011))**  **Swine Anteromedial ACL, Posterolateral ACL and ACL; v = 19,8 mm/min (Zhou et al. (2009))**  **Swine ACL and PCL; v = 19.8 mm/min (Hirokawa and Sakoshita (2003))**  **Rat cervical FCL; v = 0.08 mm/min (Quinn and Winkelstein (2007))**  **Rat FCL; v = 4,8 mm/min (Lee et al. (2006))**  **Rat MCL; v = 30 mm/min (Su et al. (2008))**  **Rabbit MCL; v = 10 mm/s (Woo et al. (1992))**  **Rabbit MCL; v = 10 mm/min (Weiss et al. (1991))**  **Rabbit MCL (female, 36 and 12 mo); v = 10 mm/min (Woo et al. (1990c))**  **Rabbit MCL (Male, 36 mo); v = 10 mm/min (Woo et al. (1990c))**  **Sheep ACL; v = 5 mm/min (Gurlek et al. (2017))**  **Sheep ACL (Right and left); v = 500 mm/min (Rogers et al. (1990))**  **Monkey ACL; v = 508,2 mm/min (Noyes et al. (1974))**  **Dog ACL; v = 1000 mm/min (Comerford et al. (2005))**  **Dog CraCL; v = 1000 mm/min (Wingfield et al. (2000))**  **Dog ACL; v = 510 mm/min (Figgie et al. (1986))** |
| **RL; v = 5 mm/min (Martins et al. (2013))** | **Swine CL (right); v = 45 mm/min (Tan et al. (2015))**  **Monkey RL; v = 6 mm/min (Vardy et al. (2005))** | **Monkey RL; v = 6 mm/min (Vardy et al. (2005))**  **Swine USL; v = 45 mm/min (Tan et al. (2015))** | **na** |
| **ALL/PLL (mean); v = 19,8 mm/min (Przybylski et al. (1996))** | **Dog ACL; v = 1000 mm/min (Comerford et al. (2005))**  **Swine USL; v = 45 mm/min (Tan et al. (2015))**  **Swine Posterolateral ACL, Anteromedial ACL and ACL; v = 19,8 mm/min (Zhou et al. (2009))**  **Swine ACL and PCL; v = 19,8 mm/min (Hirokawa and Sakoshita (2003))** | **na** | **na** |
| **USL; v = 5 mm/min (Martins et al. (2013))** | **na** | **na** | **na** |
| **PB-IGHL; v = 10 mm/min (Moore et al. (2005))** | **Swine USL; v = 45 mm/min (Tan et al. (2015))** | **na** | **Swine USL and CL (Right and left); v = 45 mm/min (Tan et al. (2015))**  **Swine MCL; v = 20 mm/min (Germscheid et al. (2011))**  **Swine Anteromedial ACL, Posterolateral ACL and ACL; v = 19,8 mm/min (Zhou et al. (2009))**  **Swine ACL and PCL; v = 19.8 mm/min (Hirokawa and Sakoshita (2003))**  **Rat FCL; v = 4,8 mm/min (Lee et al. (2006))**  **Rat MCL; v = 30 mm/min (Su et al. (2008))**  **Rabbit MCL; v = 10 mm/s (Woo et al. (1992))**  **Rabbit MCL; v = 10 mm/min (Weiss et al. (1991))**  **Rabbit MCL (female, 36 and 12 mo); v = 10 mm/min (Woo et al. (1990c))** |
| **IGHL (older); v = 50 mm/min (Lee et al. (1999))** | **Dog ACL; v = 1000 mm/min (Comerford et al. (2005))**  **Swine PCL; v = 19.8 mm/min (Hirokawa and Sakoshita (2003))** | **Swine Anteromedial ACL, Posterolateral ACL and ACL; v = 19,8 mm/min (Zhou et al. (2009))**  **Swine PCL and ACL; v = 19.8 mm/min (Hirokawa and Sakoshita (2003))** | **Swine Anteromedial ACL; v = 19,8 mm/min (Zhou et al. (2009))**  **Rabbit MCL; v = 10mm/min (Moon et al. (2006))**  **Rabbit MCL; v = 10 mm/min (Weiss et al. (1991))**  **Rabbit MCL (female, 12 and 6 mo, and male 6 mo); v = 10 mm/min (Woo et al. (1990c))** |
| **IGHL (younger); v = 50 mm/min (Lee et al. (1999))** | **Swine ACL; v = 19,8 mm/min (Hirokawa and Sakoshita (2003))**  **Swine Posterolateral ACL, Anteromedial ACL and ACL; v = 19,8 mm/min (Zhou et al. (2009))** | **na** | **Swine USL; v = 45 mm/min (Tan et al. (2015))**  **Swine MCL; v = 20 mm/min (Germscheid et al. (2011))**  **Swine Anteromedial ACL; v = 19,8 mm/min (Zhou et al. (2009))**  **Swine ACL; v = 19.8 mm/min (Hirokawa and Sakoshita (2003))**  **Rat MCL; v = 30 mm/min (Su et al. (2008))** |
| **PF; v = 5 mm/min (Pieroh et al. (2016))** | **Dog ACL; v = 1000 mm/min (Comerford et al. (2005))**  **Swine USL; v = 45 mm/min (Tan et al. (2015))** | **na** | **Rat cervical FCL; v = 0.08 mm/min (Quinn and Winkelstein (2007))**  **Dog ACL; v = 500 mm/min (Shino et al. (1984))** |
| **IS; v = 5 mm/min (Pieroh et al. (2016))** | **Swine USL; v = 45 mm/min (Tan et al. (2015))** | **na** | **Rat cervical FCL; v = 0.08 mm/min (Quinn and Winkelstein (2007))**  **Dog ACL; v = 500 mm/min (Shino et al. (1984))** |
| **IL; v = 5 mm/min (Pieroh et al. (2016))** | **Swine USL; v = 45 mm/min (Tan et al. (2015))** | **na** | **Rat cervical FCL; v = 0.08 mm/min (Quinn and Winkelstein (2007))**  **Dog ACL; v = 500 mm/min (Shino et al. (1984))** |
| **PF; v = 20 mm/min (Schleifenbaum et al. (2016))** | **Swine USL; v = 45 mm/min (Tan et al. (2015))** | **na** | **Swine CL (Right and left); v = 45 mm/min (Tan et al. (2015))**  **Swine DL; v = 2 mm/min (Polak et al. (2014))**  **Swine Anteromedial ACL, Posterolateral and ACL; v = 19,8 mm/min (Zhou et al. (2009))**  **Rat cervical FCL; v = 0.08 mm/min (Quinn and Winkelstein (2007))**  **Sheep ACL (Right and left); v = 500 mm/min (Rogers et al. (1990))**  **Monkey ACL; v = 508,2 mm/min (Noyes et al. (1974))**  **Dog ACL; v = 1000 mm/min (Comerford et al. (2005))** |
| **IS; v = 20 mm/min (Schleifenbaum et al. (2016))** | **Swine USL; v = 45 mm/min (Tan et al. (2015))** | **na** | **Swine DL; v = 2 mm/min (Polak et al. (2014))**  **Rat cervical FCL; v = 0.08 mm/min (Quinn and Winkelstein (2007))**  **Sheep ACL (Right and left); v = 500 mm/min (Rogers et al. (1990))**  **Monkey ACL; v = 508,2 mm/min (Noyes et al. (1974))**  **Dog ACL; v = 510 mm/min (Figgie et al. (1986))**  **Dog ACL; v = 500 mm/min (Shino et al. (1984))** |
| **IL; v = 20 mm/min (Schleifenbaum et al. (2016))** | **Swine USL; v = 45 mm/min (Tan et al. (2015))** | **Swine Anteromedial ACL; v = 19,8 mm/min (Zhou et al. (2009))** | **Swine DL; v = 2 mm/min (Polak et al. (2014))**  **Swine Anteromedial ACL; v = 19,8 mm/min (Zhou et al. (2009))**  **Rat cervical FCL; v = 0.08 mm/min (Quinn and Winkelstein (2007))**  **Rat FCL; v = 4,8 mm/min (Lee et al. (2006))**  **Sheep ACL; v = 5 mm/min (Gurlek et al. (2017))**  **Sheep ACL (Right and left); v = 500 mm/min (Rogers et al. (1990))**  **Monkey ACL; v = 508,2 mm/min (Noyes et al. (1974))**  **Dog ACL; v = 1000 mm/min (Comerford et al. (2005))**  **Dog ACL; v = 510 mm/min (Figgie et al. (1986b))**  **Dog ACL; v = 500 mm/min (Shino et al. (1984))** |
| **Posterior MFL; v = 200 mm/s (Gupte et al. (2002))** | **Dog CraCL; v = 1000 mm/min (Wingfield et al. (2000))**  **Sheep ACL; v = 6 mm/min (Meller et al. (2008))**  **Sheep ACL (Right and Left); v = 500 mm/min (Rogers et al. (1990))**  **Rat MCL; v = 30 mm/min (Su et al. (2008))**  **Swine MCL; v = 20 mm/min (Germscheid et al. (2011))**  **Swine PCL; v = 19.8 mm/min (Hirokawa and Sakoshita (2003))** | **na** | **na** |
| **Anterior MFL; v = 200 mm/s (Gupte et al. (2002))** | **Dog MCL; v = 20 mm/min (Woo et al. (1990b))**  **Sheep ACL; v = 6 mm/min (Meller et al. (2008))**  **Sheep ACL (Right and Left); v = 500 mm/min (Rogers et al. (1990))**  **Rabbit MCL (female, 3.5 and 6 mo) (male, 5.5 mo); v = 10 mm/min (Woo et al. (1990))**  **Rabbit Medial and Lateral ACL; v = 10 mm/min (Woo et al. (1992))**  **Rat MCL; v = 30 mm/min (Su et al. (2008))**  **Swine MCL; v = 20 mm/min (Germscheid et al. (2011))**  **Swine Posterolateral ACL, Anteromedial ACL and ACL; v = 19,8 mm/min (Zhou et al. (2009))**  **Swine PCL and ACL; v = 19.8 mm/min (Hirokawa and Sakoshita (2003))** | **na** | **na** |
| **MFL; v = 200 mm/s (Kusayama et al. (1994))** | **Dog MCL; v = 20 mm/min (Woo et al. (1990b))**  **Dog CraCL; v = 1000 mm/min (Wingfield et al. (2000))**  **Sheep ACL (Right and Left); v = 500 mm/min (Rogers et al. (1990))**  **Sheep ACL; v = 6 mm/min (Meller et al. (2008))**  **Goat MCL; v = 10 mm/min (Abramowitch et al.(2003))**  **Rabbit MCL (female, 3.5 and 6 mo) (male, 5.5 mo); v = 10 mm/min (Woo et al. (1990))**  **Rabbit Medial and Lateral ACL; v = 10 mm/min (Woo et al. (1992))**  **Rat MCL; v = 30 mm/min (Su et al. (2008))**  **Swine MCL; v = 20 mm/min (Germscheid et al. (2011))**  **Swine PCL and ACL; v = 19.8 mm/min (Hirokawa and Sakoshita (2003))** | **na** | **na** |
| **PFL; v = 6000 mm/min (La Prade et al. (2005))** | **Swine USL; v = 45 mm/min (Tan et al. (2015))** | **Swine Anteromedial ACL, Posterolateral ACL and ACL; v = 19,8 mm/min (Zhou et al. (2009))** | **na** |
| **Antero-lateral PCL; v = 1000 mm/min (Race and Amis (1994))** | **Dog CraCL; v = 1000 mm/min (Wingfield et al. (2000))**  **Sheep ACL (Right and Left); v = 500 mm/min (Rogers et al. (1990))**  **Swine Posterolateral ACL, Anteromedial ACL and ACL; v = 19,8 mm/min (Zhou et al. (2009))**  **Swine PCL and ACL; v = 19.8 mm/min (Hirokawa and Sakoshita (2003))**  **Rat MCL v=30 mm/min (Su et al.(2008))** | **Dog ACL; v = 1000 mm/min (Comerford et al. (2005))**  **Sheep ACL; v = 60 mm/min (Hunt et al. (2005))**  **Sheep ACL; v = 60 mm/min (Hunt et al. (2005))**  **Sheep ACL; v = 60 mm/min (Weiler et al. (2003))**  **Sheep ACL; v = 60 mm/min (Weiler et al. (2001))**  **Rat MCL; v = 30 mm/min (Su et al. (2008))**  **Swine Posterolateral ACL and ACL; v = 19,8 mm/min (Zhou et al. (2009))**  **Swine PCL and ACL; v = 19.8 mm/min (Hirokawa and Sakoshita (2003))**  **Swine MCL; v = 20 mm/min (Germscheid et al. (2011))** | **na** |
| **Postero-medial PCL; v = 1000 mm/min (Race and Amis (1994))** | **Dog ACL; v = 1000 mm/min (Comerford et al. (2005))**  **Dog CraCL; v = 1000 mm/min (Wingfield et al. (2000))**  **Sheep ACL (Right and Left); v = 500 mm/min (Rogers et al. (1990))**  **Swine Posterolateral ACL, Anteromedial ACL and ACL; v = 19,8 mm/min (Zhou et al. (2009))**  **Swine PCL and ACL; v = 19.8 mm/min (Hirokawa and Sakoshita (2003))** | **Dog ACL; v = 1000 mm/min (Comerford et al. (2005))**  **Swine PCL and ACL; v = 19.8 mm/min (Hirokawa and Sakoshita (2003))**  **Swine Anteromedial ACL, Posterolateral ACL and ACL; v = 19,8 mm/min (Zhou et al. (2009))** | **na** |
| **Cal (older); v = 100 mm/min (Fremerey et al. (2000))** | **na** | **Dog ACL; v = 1000 mm/min (Comerford et al. (2005))**  **Sheep ACL; v = 5 mm/min (Gurlek et al. (2017))**  **Rat MCL; v = 30 mm/min (Su et al. (2008))**  **Swine Anteromedial ACL, Posterolateral ACL and ACL; v = 19,8 mm/min (Zhou et al. (2009))**  **Swine PCL and ACL; v = 19.8 mm/min (Hirokawa and Sakoshita (2003))** | **Swine USL and Cl (Right and left); v = 45 mm/min (Tan et al. (2015))**  **Rat MCL; v = 30 mm/min (Su et al. (2008))**  **Swine Anteromedial ACL, Posterolateral ACL and ACL; v = 19,8 mm/min (Zhou et al. (2009))**  **Swine PCL and ACL; v = 19.8 mm/min (Hirokawa and Sakoshita (2003))**  **Rat MCL; v = 30 mm/min (Su et al. (2008))** |
| **Cal (younger); v = 100 mm/min (Fremerey et al. (2000))** | **na** | **Dog ACL; v = 1000 mm/min (Comerford et al. (2005))**  **Sheep ACL; v = 5 mm/min (Gurlek et al. (2017))**  **Rat MCL; v = 30 mm/min (Su et al. (2008))**  **Swine Posterolateral ACL and ACL; v = 19,8 mm/min (Zhou et al. (2009))**  **Swine PCL and ACL; v = 19.8 mm/min (Hirokawa and Sakoshita (2003))** | **Swine USL and Cl (Right); v = 45 mm/min (Tan et al. (2015))**  **Swine MCL; v = 20 mm/min (Germscheid et al. (2011))**  **Swine Posterolateral ACL; v = 19,8 mm/min (Zhou et al. (2009))**  **Swine ACL; v = 19,8 mm/min (Hirokawa and Sakoshita (2003))** |
| **AB-IGHL/PB-IGHL/SB-IGHL (mean); v = 2,4mm/min (Bigliani et al. (1992))** | **na** | **na** | **Swine Cl (Right and left); v = 45 mm/min (Tan et al. (2015))**  **Swine MCL; v = 20 mm/min (Germscheid et al. (2011))**  **Swine Anteromedial ACL, Posterolateral ACL and ACL; v = 19,8 mm/min (Zhou et al. (2009))**  **Swine ACL; v = 19,8 mm/min (Hirokawa and Sakoshita (2003))**  **Rat MCL; v = 30 mm/min (Su et al. (2008))**  **Rat FCL; v = 4,8 mm/min (Lee et al. (2006))** |
| **FAL; v = 2.4 mm/s (Hewitt et al. (2002))** | **na** | **na** | **Swine USL and CL (right); v = 45 mm/min (Tan et al. (2015))**  **Swine MCL; v = 20 mm/min (Germscheid et al. (2011))**  **Swine Anteromedial ACL; v = 19,8 mm/min (Zhou et al. (2009))**  **Swine PCL and ACL; v = 19.8 mm/min (Hirokawa and Sakoshita (2003))**  **Rat MCL; v = 30 mm/min (Su et al. (2008))**  **Rabbit MCL; v = 10mm/min (Moon et al. (2006))**  **Rabbit MCL; v = 10 mm/s (Woo et al. (1992b))**  **Rabbit MCL; v = 10 mm/min (Weiss et al. (1991))**  **Rabbit MCL (female, 6, 12 and 36 mo) (male, 6, 12 and 36 mo); v = 10 mm/min (Woo et al. (1990c))** |
| **IS; v = 2.4 mm/min (Hewitt et al. (2002))** | **na** | **Monkey USL; v = 6 mm/min (Vardy et al. (2005))**  **Swine CL (left and right); v = 45 mm/min (Tan et al. (2015))** | **na** |
| **SHIL; v = 2,4 mm/min (Hewitt et al. (2002))** | **na** | **Monkey RL; v = 6 mm/min (Vardy et al. (2005))**  **Swine USL; v = 45 mm/min (Tan et al. (2015))**  **Swine DL; v = 2 mm/min (Polak et al. (2014))** | **Swine Anteromedial ACL; v = 19,8 mm/min (Zhou et al. (2009))**  **Rabbit MCL; v = 10mm/min (Moon et al. (2006))**  **Rabbit MCL (female and male, 6 mo); v = 10 mm/min (Woo et al. (1990))** |
| **IHIL; v = 2.4 mm/min (Hewitt et al. (2002))** | **na** | **na** | **Swine Anteromedial ACL; v = 19,8 mm/min (Zhou et al. (2009))**  **Swine PCL and ACL; v = 19.8 mm/min (Hirokawa and Sakoshita (2003))**  **Rabbit MCL; v = 10mm/min (Moon et al. (2006))**  **Rabbit MCL; v = 10 mm/s (Woo et al. (1992b))**  **Rabbit MCL; v = 10 mm/min (Weiss et al. (1991))**  **Rabbit MCL (female, 6, 12 and 36 mo) (male, 6, 12 and 36 mo); v = 10 mm/min (Woo et al. (1990c))** |
| **Scapholunate Ligament; v = 50 mm/min (Johnston et al. (2004))** | **na** | **na** | **Swine DL; v = 2 mm/min (Polak et al. (2014))**  **Rat cervical FCL; v = 0.08 mm/min (Quinn and Winkelstein (2007))**  **Rat FCL; v = 4,8 mm/min (Lee et al. (2006))**  **Sheep ACL; v = 5 mm/min (Gurlek et al. (2017))**  **Sheep ACL (Right and left); v = 500 mm/min (Rogers et al. (1990))**  **Monkey ACL; v = 508,2 mm/min (Noyes et al. (1974))**  **Dog ACL; v = 510 mm/min (Figgie et al. (1986))** |
| **PFL; v = 200 mm/min (Sugita and Amis (2001a))** | **na** | **na** | **Swine USL and CL (Right); v = 45 mm/min (Tan et al. (2015))**  **Swine MCL; v = 20 mm/min (Germscheid et al. (2011))**  **Swine Anteromedial ACL; v = 19,8 mm/min (Zhou et al. (2009))**  **Swine ACL; v = 19.8 mm/min (Hirokawa and Sakoshita (2003))**  **Rat MCL; v = 30 mm/min (Su et al. (2008))**  **Rabbit MCL; v = 10 mm/s (Woo et al. (1992b))**  **Rabbit MCL; v = 10 mm/min (Weiss et al. (1991))**  **Rabbit MCL (female, 12 and 36 mo, and male 36 mo); v = 10 mm/min (Woo et al. (1990))** |
| **LCL; v = 200 mm/min (Sugita and Amis (2001a))** | **na** | **na** | **Swine USL; v = 45 mm/min (Tan et al. (2015))**  **Swine MCL; v = 20 mm/min (Germscheid et al. (2011))**  **Swine Anteromedial ACL; v = 19,8 mm/min (Zhou et al. (2009))**  **Swine ACL; v = 19.8 mm/min (Hirokawa and Sakoshita (2003))**  **Rat MCL; v = 30 mm/min (Su et al. (2008))**  **Rabbit MCL; v = 10 mm/s (Woo et al. (1992b))**  **Rabbit MCL; v = 10 mm/min (Weiss et al. (1991))** |
