# Supplementary material for: Mechanical properties of animal ligaments: a review and comparative study for the identification of the most suitable human ligament surrogates
Source: Biomech Model Mechanobiol. 2023 May 11;22(5):1645–83. doi: 10.1007/s10237-023-01718-1 (PMC10511400; doi:10.1007/s10237-023-01718-1)
Supplement: Supplementary file 2 — Supplementary file2 (DOCX 33 kb) [file 10237_2023_1718_MOESM2_ESM.docx]

| **Reference** | **Elastic Modulus** | **Ultimate Stress** | **Ultimate strain** |
| --- | --- | --- | --- |
| **ACL; v=6000 %/min (Noyes and Grood (1976))** | **na** | **Calf LCL; v=60 %/min (Eleswarapu et al. (2011))**  **Calf MCL; v=60 %/min (Eleswarapu et al. (2011))**  **Calf CauCL; v=60%/min (Eleswarapu et al. (2011))** | **na** |
| **ACL; v=6000 %/min (Chandrashekar et al. (2006))** | **Sheep ACL; v=300%/min (Mahalingam et al. (2015))**  **Sheep Antero-medial ACL; v=300%/min (Mallett and Arruda (2017))** | **Calf LCL and MCL; v=60%/min (Eleswarapu et al. (2011))** | **Equine SL; v=4800 %/min (Smith (2006))**  **Goat ACL; v=6000%/min (Jackson et al. (1991))** |
| **ACL; v=6000 %/min (Chandrashekar et al. (2006))** | **Monkey ACL; v=3960%/min (Noyes and Grood (1976))**  **Sheep ACL; v=300%/min (Mahalingam et al. (2015))**  **Sheep Antero-medial ACL; v=300%/min (Mallett and Arruda (2017))** | **Calf LCL; v=60%/min (Eleswarapu et al. (2011))** | **Goat ACL; v=6000%/min (Jackson et al. (1991))** |
| **Antero later PCL; v=3000 %/min (Race and Amis (1994))** | **Sheep Antero-medial ACL; v=300 %/min (Mallett and Arruda (2017))**  **Swine LCL; v=60 %/min (Bonner et al. (2015))**  **Goat ACL; v=4800%/min (Ng et al. (1995))**  **Goat ACL; v=6000%/min (Jackson et al. (1993))**  **Monkey (Noyes and Grood (1976))**  **Dog AMCL-V; v=30%/min (Woo et al. (1990b))**  **Monkey ACL; v=3960%/min (Noyes and Grood (1976))**  **Sheep ACL; v=300%/min (Mahalingam et al. (2015))** | **Swine LCL; v=60 %/min (Bonner et al. (2015))**  **Swine LCL; v=600 %/min (Bonner et al. (2015))** | **Equine SL, v=60%/min (Riemersma and Schamhardt (1985))**  **Equine SL; v=4800 %/min (Smith (2006))**  **Swine LCL; v=60 %/min (Bonner et al. (2015))**  **Swine LCL; v=600 %/min (Bonner et al. (2015))**  **Swine LCL; v=5640 %/min (Bonner et al. (2015))**  **Equine AccL; v=60-180 %/min (Becker et al. (1994))** |
| **Postero medial PCL; v=3000 %/min (Race and Amis (1994))** | **Dog AMCL-V; v=30%/min (Woo et al. (1990b))**  **Monkey ACL; v=3960%/min (Noyes and Grood (1976))**  **Sheep ACL; v=300%/min (Mahalingam et al. (2015))**  **Swine LCL; v=60 %/min (Bonner et al. (2015))**  **Sheep Antero-medial ACL; v=300%/min (Mallett and Arruda (2017))** | **Swine LCL; v=60 %/min (Bonner et al. (2015))**  **Calf LCL and MCL; v=60%/min (Eleswarapu et al. (2011))** | **Equine SL; v=4800 %/min (Smith( 2006))**  **Swine LCL; v=60 %/min (Bonner et al. (2015))**  **Swine LCL; v=600 %/min (Bonner et al. (2015))**  **Swine LCL; v=5640 %/min (Bonner et al. (2015))**  **Equine AccL; v=60-180 %/min (Becker et al. (1994))** |
| **FCL; v=6000 %/min (LaPrade et al. (2005))** | **Dog AMCL-V; v=30%/min (Woo et al. (1990b))**  **Monkey ACL; v=3960%/min (Noyes and Grood (1976))**  **Goat ACL; v=6000%/min (Jackson et al. (1993))**  **Sheep ACL; v=300%/min (Mahalingam et al. (2015))**  **Sheep Antero-medial ACL; v=300%/min (Mallett and Arruda (2017))**  **Swine LCL; v=60 %/min and V=600%/min (Bonner et al. (2015))** | **Swine LCL; v=60 %/min (Bonner et al. (2015))**  **Calf LCL and MCL; v=60%/min (Eleswarapu et al. (2011))** | **na** |
| **MCL; v=60 %/min (longitudinal) (Quapp and Weiss (1997))** | **Dog AMCL-V and MCL; v=30 %/min (Woo et al. 1990b))**  **Goat ACL; v=4800 %/min (Ng et al. (1995))**  **Sheep Antero-medial ACL; v=300 %/min (Mallett and Arruda (2017))**  **Swine LCL; v=60 %/min (Bonner et al. 2015))**  **Swine LCL; v=600 %/min (Bonner et al. 2015))** | **Swine LCL; v=60 %/min (Bonner et al. (2015))** | **Equine SL; v=4800 %/min (Smith (2006))**  **Swine LCL; v=60 %/min (Bonner et al. (2015))**  **Swine LCL; v=600 %/min (Bonner et al. (2015))**  **Swine LCL; v=5640 %/min (Bonner et al. (2015))**  **Equine AccL; v=60-180 %/min (Becker et al. (1994))** |
| **MCL; v=60 %/min (transverse) (Quapp and Weiss (1997))** | **Calf CauCL, LCL and MCL; v=60 %/min (Eleswarapu et al. (2011))**  **Cow PL; v=600 %/min (Oskui et al. (2016))**  **Cow PL; v=6000 %/min (Oskui et al. (2016))**  **Cow PL; v=60000 %/min (Oskui et al. (2016))** | **Calf CraCL and CauCL; v=60 %/min (Eleswarapu et al. (2011))** | **Equine SL, v=60%/min (Riemersma and Schamhardt (1985))**  **Equine SL; v=60-180 %/min (Jansen and Savelberg (1994))**  **Equine AccL and DCL; v=60-180 %/min (Becker et al. (1994))**  **Foal AccL; v=60-180 %/min (Becker et al. (1994))**  **Rabbit MCL; v=1116±150 %/min (Woo et al. (1990a))**  **Rabbit MCL; v=27±4.8%/min (Moon et al. (2006))**  **Swine LCL; v=63600 %/min (Bonner et al. (2015))** |
| **MPFL; v=18%/min (Criscenti et al. (2016))​** | **Dog AMCL-V and MCL; v=30 %/min (Woo et al. 1990b))**  **Monkey ACL; v=3960%/min (Noyes and Grood (1976))**  **Sheep ACL; v=300%/min (Mahalingam et al. (2015))**  **Sheep Antero-medial ACL; v=300%/min (Mallett and Arruda (2017))**  **Swine LCL; v=60 %/min and V=600%/min (Bonner et al. (2015))** | **Calf CauCL, LCL and MCL; v=60 %/min (Eleswarapu et al. (2011))** | **Equine SL; v=4800 %/min (Smith( 2006))**  **Swine LCL; v=60 %/min (Bonner et al. (2015))**  **Swine LCL; v=600 %/min (Bonner et al. (2015))**  **Goat ACL; v=6000%/min (Jackson et al. (1991))** |
| **ALL; v=3000 %/min (Mattucci et al. (2012))** | **na** | **Swine LCL; v=60 %/min (Bonner et al. (2015))** | **Cow PL; v=600 %/min (Oskui et al. (2016))**  **Cow PL; v=6000 %/min (Oskui et al. (2016))**  **Cow PL; v=60000 %/min (Oskui et al. (2016))** |
| **ALL; v=12000 %/min (Mattucci et al. (2012))** | **na** | **Foal AccL; v=60-180 %/min (Becker et al. (1994))**  **Swine LCL; v=60 %/min (Bonner et al. (2015))**  **Swine LCL; v=600 %/min (Bonner et al. (2015))** | **na** |
| **ALL; v=900000 %/min (Mattucci et al. (2012))** | **na** | **Foal AccL; v=60-180 %/min (Becker et al. (1994))**  **Swine LCL; v=60 %/min (Bonner et al. (2015))** | **na** |
| **PLL; v=3000 %/min (Mattucci et al. (2012))** | **na** | **Calf LCL; v=60 %/min (Eleswarapu et al. (2011))**  **Swine LCL; v=60 %/min (Bonner et al. (2015))** | **na** |
| **PLL; v=12000 %/min (Mattucci et al. (2012))** | **na** | **Cow PL; v=60 %/min (Oskui et al. (2016))**  **Cow PL; v=600 %/min (Oskui et al. (2016))**  **Cow PL; v=6000 %/min (Oskui et al. (2016))**  **Cow PL; v=60000 %/min (Oskui et al. (2016))**  **Calf CauCL, LCL and MCL; v=60 %/min (Eleswarapu et al. (2011))**  **Dog MCL; v=30%/min (Woo et al. (1990b))**  **Equine SL; v=60-180 %/min (Jansen and Savelberg (1994))**  **Equine AccL and DCL; v=60-180 %/min (Becker et al. (1994))**  **Foal AccL; v=60-180 %/min (Becker et al. (1994))**  **Swine LCL; v=60 %/min (Bonner et al. (2015))**  **Swine LCL; v=600 %/min (Bonner et al. (2015))**  **Swine LCL; v=5640 %/min (Bonner et al. (2015))** | **Equine SL; v=4800 %/min (Smith( 2006))**  **Swine LCL; v=60 %/min (Bonner et al. (2015))**  **Swine LCL; v=600 %/min (Bonner et al. (2015))**  **Swine LCL; v=5640 %/min (Bonner et al. (2015))**  **Swine LCL; v=63600 %/min (Bonner et al. (2015))**  **Swine LCL; v=779400 %/min (Bonner et al. (2015))**  **Goat ACL; v=6000%/min (Jackson et al. (1991))**  **Equine SL, v=60%/min (Riemersma and Schamhardt (1985))**  **Equine SL; v=60-180 %/min (Jansen and Savelberg (1994))**  **Equine AccL and DCL; v=60-180 %/min (Becker et al. (1994))**  **Foal AccL; v=60-180 %/min (Becker et al. (1994))**  **Rabbit MCL; v=0.66±0.06 %/min (Woo et al. (1990a))**  **Rabbit MCL; v=** **v=9±0.6 %/min (Woo et al. (1990a))**  **Rabbit MCL; v=1116±150 %/min (Woo et al. (1990a))**  **Rabbit MCL; v=** **v=** **v=13200±1500 %/min (Woo et al. (1990a))**  **Rabbit MCL; v=27±4.8%/min (Moon et al. (2006))** |
| **PLL; v=900000 %/min (Mattucci et al. (2012))** | **Dog AMCL-V and MCL; v=30 %/min (Woo et al. 1990b))**  **Monkey ACL; v=3960%/min (Noyes and Grood (1976))**  **Sheep ACL; v=300%/min (Mahalingam et al. (2015))**  **Sheep Antero-medial ACL; v=300%/min (Mallett and Arruda (2017))**  **Swine LCL; v=60 %/min (Bonner et al. (2015))** | **Foal AccL; v=60-180 %/min (Becker et al. (1994))**  **Swine LCL; v=60 %/min (Bonner et al. (2015))**  **Swine LCL; v=600 %/min (Bonner et al. (2015))** | **na** |
| **CL; v=3000 %/min (Mattucci et al. (2012))** | **Cow PL; v=60 %/min (Oskui et al. (2016))**  **Cow PL; v=600 %/min (Oskui et al. (2016))**  **Calf CauCL and MCL; v=60 %/min (Eleswarapu et al. (2011))** | **Cow PL; v=60 %/min (Oskui et al. (2016))**  **Cow PL; v=600 %/min (Oskui et al. (2016))**  **Cow PL; v=6000 %/min (Oskui et al. (2016))**  **Cow PL; v=60000 %/min (Oskui et al. (2016))**  **Calf CauCL and MCL; v=60 %/min (Eleswarapu et al. (2011))** | **na** |
| **CL; v=12000 %/min (Mattucci et al. (2012))** | **Cow PL; v=600 %/min (Oskui et al. (2016))**  **Cow PL; v=6000 %/min (Oskui et al. (2016))**  **Calf CauCL and MCL; v=60 %/min (Eleswarapu et al. (2011))** | **Calf CauCL; v=60 %/min (Eleswarapu et al. (2011))**  **Calf MCL; v=60 %/min (Eleswarapu et al. (2011))** | **Cow PL; v=600 %/min (Oskui et al. (2016))**  **Cow PL; v=6000 %/min (Oskui et al. (2016))**  **Cow PL; v=60000 %/min (Oskui et al. (2016))** |
| **CL; v=900000 %/min (Mattucci et al. (2012))** | **Cow PL; v=600 %/min (Oskui et al. (2016))**  **Cow PL; v=6000 %/min (Oskui et al. (2016))**  **Cow PL; v=60000 %/min (Oskui et al. (2016))**  **Calf CauCL, ACLand MCL; v=60 %/min (Eleswarapu et al. (2011))** | **Calf CauCL; v=60 %/min (Eleswarapu et al. (2011))**  **Calf MCL; v=60 %/min (Eleswarapu et al. (2011))** | **Cow PL; v=6000 %/min (Oskui et al. (2016))**  **Cow PL; v=60000 %/min (Oskui et al. (2016))** |
| **LF; v=33%/min (Nachemson and Evans (1968))** | **na** | **Calf CraCL and CauCL; v=60 %/min (Eleswarapu et al. (2011))** | **na** |
| **LF; v=33%/min (Nachemson and Evans (1968))** | **na** | **Calf MCL; v=60 %/min (Eleswarapu et al. (2011))** | **na** |
| **LF; v=33%/min (Nachemson and Evans (1968))** | **na** | **Calf CraCL and CauCL; v=60 %/min (Eleswarapu et al. (2011))** | **Dog CraCL; v=6000%/min (Butler et al. (1983))**  **Goat ACL; v=6000%/min (Jackson et al. (1991))** |
| **LF; v=33%/min (Nachemson and Evans (1968))** | **na** | **Calf CraCL and CauCL; v=60 %/min (Eleswarapu et al. (2011))** | **Equine SL, v=60%/min (Riemersma and Schamhardt (1985))**  **Equine SL; v=60-180 %/min (Jansen and Savelberg (1994))**  **Equine AccL and DCL; v=60-180 %/min (Becker et al. (1994))**  **Foal AccL; v=60-180 %/min (Becker et al. (1994))**  **Swine LCL; v=60 %/min (Bonner et al. (2015))**  **Swine LCL; v=600 %/min (Bonner et al. (2015))** |
| **LF; v=33%/min (Nachemson and Evans 1968))** | **na** | **Cow PL; v=60 %/min (Oskui et al. (2016))**  **Cow PL; v=600 %/min (Oskui et al. (2016))**  **Cow PL; v=6000 %/min (Oskui et al. (2016))**  **Cow PL; v=60000 %/min (Oskui et al. (2016))**  **Calf GraCL and CauCL; v=60 %/min (Eleswarapu et al. (2011))** | **na** |
| **LF; v=33%/min (Nachemson and Evans (1968))** | **na** | **Calf CraCL and CauCL; v=60 %/min (Eleswarapu et al. (2011))** | **na** |
| **LF; v=33%/min (Nachemson and Evans (1968))** | **na** | **Calf CauCL; v=60 %/min (Eleswarapu et al. (2011))** | **Goat ACL; v=6000 %/min (Jackson et al. (1991))** |
| **LF; v=33%/min (Nachemson and Evans (1968))** | **na** | **Cow PL; v=60 %/min (Oskui et al. (2016))**  **Cow PL; v=600 %/min (Oskui et al. (2016))**  **Cow PL; v=6000 %/min (Oskui et al. (2016))**  **Calf CauCL; v=60 %/min (Eleswarapu et al. (2011))** | **Goat ACL; v=6000 %/min (Jackson et al. (1991))** |
| **LF; v=3000 %/min (Mattucci et al. (2012))** | **Cow PL; v=6000 %/min (Oskui et al. (2016))**  **Cow PL; v=60000 %/min (Oskui et al. (2016))**  **Calf CauCL, LCL and MCL; v=60 %/min (Eleswarapu**  **et al. (2011))** | **Cow PL; v=6000 %/min (Oskui et al. (2016))**  **Cow PL; v=60000 %/min (Oskui et al. (2016))**  **Calf CauCL and MCL; v=60 %/min (Eleswarapu et al. 2011))** | **na** |
| **LF; v=12000 %/min (Mattucci et al. (2012))** | **Calf CL and MCL; v=60 %/min (Eleswarapu**  **et al. (2011))** | **Calf CauCL and MCL; v=60 %/min (Eleswarapu et al. (2011))** | **na** |
| **LF; v=900000 %/min (Mattucci et al. (2012))** | **Calf CL and MCL; v=60 %/min (Eleswarapu**  **et al. (2011))** | **Calf CauCL and MCL; v=60 %/min (Eleswarapu et al. (2011))** | **na** |
| **ISL; v=3000 %/min (Mattucci et al. (2012))** | **Cow PL; v=60 %/min (Oskui et al. (2016))**  **Cow PL; v=600 %/min (Oskui et al. (2016))**  **Cow PL; v=6000 %/min (Oskui et al. (2016))**  **Cow PL; v=60000 %/min (Oskui et al. (2016))**  **Calf CauCL, LCL and MCL; v=60 %/min (Eleswarapu**  **et al. (2011))** | **Cow PL; v=60 %/min (Oskui et al. (2016))**  **Cow PL; v=600 %/min (Oskui et al. (2016))**  **Cow PL; v=6000 %/min (Oskui et al. (2016))**  **Cow PL; v=60000 %/min (Oskui et al. (2016))**  **Calf CauCL and CraCL; v=60 %/min (Eleswarapu**  **et al. (2011))** | **na** |
| **ISL; v=12000 %/min (Mattucci et al. (2012))** | **Cow PL; v=60 %/min (Oskui et al. (2016))**  **Cow PL; v=600 %/min (Oskui et al. (2016))**  **Cow PL; v=6000 %/min (Oskui et al. (2016))**  **Cow PL; v=60000 %/min (Oskui et al. (2016))**  **Calf CauCL, LCL and MCL; v=60 %/min (Eleswarapu**  **et al. (2011))** | **Cow PL; v=60 %/min (Oskui et al. (2016))**  **Cow PL; v=600 %/min (Oskui et al. (2016))**  **Cow PL; v=6000 %/min (Oskui et al. (2016))**  **Cow PL; v=60000 %/min (Oskui et al. (2016))**  **Calf CauCL, CraCL, LCL and MCL; v=60 %/min (Eleswarapu et al. 2011))** | **Dog CraCL; v=6000%/min (Butler et al. (1983))**  **Goat ACL; v=6000 %/min (Jackson et al. (1991))** |
| **ISL; v=900000 %/min (Mattucci et al. (2012))** | **Cow PL; v=60 %/min (Oskui et al. (2016))**  **Cow PL; v=600 %/min (Oskui et al. (2016))**  **Cow PL; v=6000 %/min (Oskui et al. (2016))**  **Cow PL; v=60000 %/min (Oskui et al. (2016))**  **Calf CauCL, LCL and MCL; v=60 %/min (Eleswarapu**  **et al. (2011))** | **Cow PL; v=60 %/min (Oskui et al. (2016))**  **Cow PL; v=600 %/min (Oskui et al. (2016))**  **Cow PL; v=6000 %/min (Oskui et al. (2016))**  **Cow PL; v=60000 %/min (Oskui et al. (2016))**  **Calf CauCL, CraCL, LCL and MCL; v=60 %/min (Eleswarapu et al. 2011))** | **Dog CraCL; v=6000%/min (Butler et al. (1983))**  **Goat ACL; v=6000 %/min (Jackson et al. (1991))** |
